# Effectiveness of Therapeutic Exercise in Reducing the Severity of Primary Dysmenorrhea and Associated Symptoms: A Systematic Review and Meta-Analysis

**DOI:** 10.3390/jcm15124418

**Published:** 2026-06-07

**Authors:** Rubén Arroyo-Fernández, Cristina Lirio-Romero, Claudia A. Quezada-Bascuñán, Igor Cigarroa, Emanuele Marzetti, Elisabeth Bravo-Esteban

**Affiliations:** 1 Faculty of Physiotherapy and Nursing, University of Castilla-La Mancha, 45071 Toledo, Spain; ruben.arroyo@uclm.es (R.A.-F.); claudia.quezada@uclm.es (C.A.Q.-B.); elisabeth.bravo@uclm.es (E.B.-E.); 2 Pain, Mental Health, Exercise and Technology Research Group (PAIN+MET), Instituto de Investigación Sanitaria de Castilla-La Mancha (IDISCAM), 45071 Toledo, Spain; 3 Hospital General Universitario Nuestra Señora del Prado, Gerencia de Atención Integrada de Talavera de la Reina (SESCAM), 45600 Talavera de la Reina, Spain; 4 ImproveLab Research Group in Paediatric Physiotherapy and Neurology, Instituto de Investigación Sanitaria de Castilla-La Mancha (IDISCAM), 45071 Toledo, Spain; 5 Health and Social Research Center, University of Castilla-La Mancha, 16002 Cuenca, Spain; 6 Facultad de Ciencias de la Salud, Universidad Arturo Prat, Victoria 4720000, Chile; icigarroac@ucsh.cl; 7 Department of Geriatrics, Orthopedics and Rheumatology, Fondazione Policlinico Universitario “A. Gemelli” IRCCS, Università Cattolica del Sacro Cuore, 00168 Rome, Italy; emanuele.marzetti@unicatt.it; 8 Physiotherapy Research Group, Toledo (GIFTO), Instituto de Investigación Sanitaria de Castilla-La Mancha (IDISCAM), 45071 Toledo, Spain

**Keywords:** primary dysmenorrhea, pain, exercise, quality of life, symptom severity

## Abstract

**Background and objectives:** Primary dysmenorrhea is a major contributor to chronic pelvic pain. It negatively impacts quality of life. Therapeutic exercise has become recognized as a non-pharmacological alternative for reducing the severity of symptoms. **Methods:** A systematic review and meta-analysis were conducted in accordance with the PRISMA guidelines. Randomized controlled trials involving adult women with primary dysmenorrhea who underwent therapeutic exercise interventions were selected. Databases searched were Embase, PubMed, Scopus, CENTRAL, Web of Science, ProQuest, PEDro, and CINAHL up to 31 January 2026. The main variables were symptom severity, pain intensity, and duration. The secondary outcomes were quality of life, anxiety, and sleep quality. The Cochrane’s Risk of Bias 2 tool was used to assess the risk of bias, while the certainty of evidence was determined using GRADE. **Results:** Twenty-nine studies (1704 participants) were included. Therapeutic exercise significantly improved symptom severity (SMD = −0.91), pain intensity (SMD = −2.17), and duration (SMD = −0.70), and enhanced quality of life (SMD = 0.70) and sleep quality (SMD = 0.33). These results were maintained into the second menstrual cycle after the intervention. I^2^ values were high across all outcomes. Subgroup analysis revealed a greater effect of mind–body and stretching interventions on symptom severity, and of aerobic and strength exercise on pain intensity. The certainty of the evidence was low to very low. **Conclusions:** Therapeutic exercise appears effective for improving the severity of primary dysmenorrhea and associated symptoms, although caution is warranted when interpreting these conclusions due to the insufficient certainty of the evidence.

## 1. Introduction

Primary dysmenorrhea (PD) is defined by recurrent, cramping pain localized in the pelvic region or lower abdomen that begins shortly before or at the onset of menstruation. Unlike secondary dysmenorrhea, PD occurs in the absence of identifiable pelvic pathology. It may be associated with systemic symptoms such as fatigue, mood and sleep disturbances, nausea, headache, and gastrointestinal discomfort [1]. The World Health Organization considers PD the leading contributor to chronic pelvic pain, defined as persistent or recurrent, cyclic or non-cyclic pain located in the lower abdomen or pelvis and lasting at least 3 months. Factors such as being younger than 25 years of age or living in less developed countries appear to be determinants for its occurrence [2,3]. Its global prevalence varies considerably, although it is estimated to affect approximately 71% of women of reproductive age [1].

Recent studies have highlighted the negative impact of PD on quality of life, social life, and professional development, and have identified it as a common cause of school or work absenteeism [4,5]. However, pain is not the only relevant factor contributing to reduced quality of life in affected women. Some authors have reported a close relationship between sleep quality and psychological factors such as anxiety in the days preceding and during menstruation, which is associated with greater severity of PD symptoms [6,7].

International expert panels strongly recommend therapeutic exercise (TE) for improving the severity of PD symptoms [8]. The mechanisms underlying the effects of TE are primarily related to the secretion of endorphins, cannabinoids, and anti-inflammatory macrophages, thereby reducing pain sensitivity and inhibiting the activity of pro-inflammatory factors [9,10]. In addition, TE promotes the release of neurotransmitters such as dopamine and serotonin, which help promote well-being and alleviate psychological symptoms [11].

Previous systematic reviews have suggested that TE may help improve PD symptoms. However, most have focused exclusively on the effect of TE on pain [11,12,13] and symptom severity [14]. Unlike previous reviews, the present study included only adult women, as PD in adolescents may be influenced by the immaturity of the hypothalamic–pituitary–ovarian axis and the menstrual irregularity characteristic of the early post-menarche years [15]. Notably, this review also examines the effects of TE on quality of life and psychosocial variables such as anxiety and sleep quality, which have not been assessed in previous systematic reviews despite their strong correlation with reductions in PD symptom severity [6,7].

Therefore, this systematic review and meta-analysis aimed to evaluate the effectiveness of TE in women with PD. The primary outcomes were symptom severity and pain intensity and duration. Secondary outcomes included quality of life, anxiety, and sleep quality.

## 2. Materials and Methods 

### 2.1. Protocol

This study is a systematic review and meta-analysis conducted following the Preferred Reporting Items for Systematic Reviews and Meta-Analyses (PRISMA) guidelines [16] (Supplementary S1) and the methodological standards recommended by the Cochrane Collaboration [17]. The protocol was prospectively registered in the International Prospective Register of Systematic Reviews (PROSPERO; registration number: CRD420251269854). No significant deviations from the registered protocol occurred during the review process.

### 2.2. Data Sources and Searches

Two investigators (EBE and CLR) independently conducted a comprehensive literature search in the following electronic databases: Embase, MEDLINE (via PubMed), Scopus, CENTRAL (Cochrane Central Register of Controlled Trials), Web of Science, ProQuest, PEDro, and CINAHL. Studies published in any language from database inception to 31 January 2026 were considered. The search aimed to identify randomized controlled trials (RCTs) evaluating TE interventions in women with PD.

The search terms included primary dysmenorrhea, therapeutic exercise, symptom severity, pain, quality of life, anxiety, and sleep quality. Boolean operators (AND/OR) and both free-text terms and Medical Subject Headings (MeSH) were linked as appropriate. Supplementary S1 contains the exhaustive search protocols for all screened databases.

A specialized medical librarian was consulted to review the quality and completeness of the search strategy. A manual check of the bibliographies of all eligible papers was performed to capture any further relevant publications. Additionally, the clinical trial registry databases of the National Library of Medicine (https://clinicaltrials.gov/; accessed on 15 January 2025) and the European Medicines Agency (https://www.clinicaltrialsregister.eu/; accessed on 15 January 2025) were consulted. A third investigator (RAF) intervened to facilitate consensus in the event of any non-concurrence between the primary reviewers.

### 2.3. Study Selection

Following title and abstract screening, two investigators (EBE and CLR) independently assessed the full texts of potentially eligible studies. Studies were selected if they evaluated the effectiveness of TE on the severity of PD and its associated symptoms, according to the PICOS framework: (i) participants: adult women with PD (from 18 to 45 years old); (ii) intervention: TE; (iii) comparator: no intervention; (iv) outcomes: symptom severity, pain intensity and duration, quality of life, anxiety, and sleep quality; and (v) study design: RCTs only.

The inclusion of different types of TE (aerobic, strength, stretching, mind–body, or multimodal) reflects the primary objective of evaluating TE as a global clinical entity. The intrinsic variability of the protocols was appropriately addressed through statistical analyses stratified by exercise type, allowing for a precise and clinically meaningful interpretation without compromising the overall effect of TE.

The selection of study variables adhered to the principles of the COMET (Core Outcome Measures in Effectiveness Trials) initiative [18]. This initiative is based on incorporating the perspectives of individuals diagnosed with the condition of interest through qualitative methods, with the aim of identifying outcomes that are relevant and meaningful to patients, as recommended in the development of core outcome sets. Thus, symptom severity and pain intensity and duration were agreed upon as the core domains in PD by Delphi groups composed of experts and healthcare professionals [19]. Therefore, the severity of PD symptoms and the intensity and duration of pain were considered as the main variables of the review, to which quality of life, anxiety, and sleep quality were added as secondary variables.

Exclusion criteria were as follows: quasi-experimental or case-crossover designs; studies available only as abstracts or conference proceedings; studies that included pharmacological treatment or the intake of nutritional supplements; studies in women with other conditions that do not stratify data by specific diagnosis; and studies that failed to provide adequate quantitative results for the predefined endpoints. Disagreements were resolved through consultation with a third investigator (RAF).

### 2.4. Data Extraction

Data extraction was performed independently by two investigators (CQB and CLR) using a standardized Excel spreadsheet specifically developed for this study. The following information was extracted from each included study:

(i) study characteristics: first author, year of publication, study location, sample size, and mean age of participants; (ii) intervention characteristics: type of exercise, weekly frequency and total duration, and session duration; (iii) outcome variables: type of outcome and assessment tool; and (iv) dropouts. Authors of the selected studies were contacted to obtain or clarify missing or unclear data if needed. Data available only in graphs [20] were extracted using the WebPlotDigitizer v.5 software for graph digitalization (https://automeris.io/ (accessed on 3 February 2026)).

### 2.5. Risk of Bias and Certainty of Evidence Assessment

The methodological quality of included studies was assessed using the Cochrane Risk of Bias 2 (RoB 2) tool [21] by two independent researchers (IC and EM). A third investigator (CLR) resolved any disagreements. This tool evaluates five domains: the randomization process, deviations from intended interventions, missing outcome data, measurement of the outcome, and selection of the reported results. Each domain, as well as the overall risk of bias, was classified as low risk, some concerns, or high risk.

The certainty of the evidence was assessed using the Grading of Recommendations Assessment, Development and Evaluation (GRADE) approach [22]. Two independent investigators (IC and EM) conducted the assessments, with disagreements resolved by a third investigator (CLR).

### 2.6. Statistical Methods

The inverse variance method was applied to analyze all variables. Heterogeneity was examined using the Chi-square test (*p* < 0.10) and quantified with the I^2^ statistic [17].

Based on the observed I^2^ statistic, we utilized either a random-effects (I^2^ > 50%) or a fixed-effects (I^2^ ≤ 50%) approach. When studies utilized consistent measurement scales, MDs were estimated; otherwise, SMDs were used to account for differences across scoring systems. In the pooled analyses, positive values expressed in the forest plots consistently indicated a benefit in favor of TE. For rating scales where a higher score corresponds to lower symptom severity (SF-36), this value was multiplied by −1 to align the direction of the effect. All results were presented with 95% confidence intervals (CIs). Data corresponding to the final values of the two menstrual cycles following the intervention were included in the quantitative synthesis.

For trials featuring multiple treatment arms, shared control participants were partitioned to avoid artificial inflation of the sample size [17]. Subgroup analyses were conducted according to the type of TE intervention (aerobic exercise, strength, stretching, mind–body interventions, and multimodal interventions), provided that at least two studies were available per subgroup. A secondary subgroup analysis was performed for the main variables based on the duration of the interventions (≤4 weeks, between 5 and 8 weeks, ≥9 weeks), type of assessment tool used, and risk of bias. Statistical processing was performed utilizing Review Manager version 5.4.1 (Copenhagen, Denmark).

The safety and acceptability of the intervention were assessed by analyzing dropout rates, using the Mantel–Haenszel method to calculate the risk ratio.

Sensitivity analyses were performed by omitting individual studies to evaluate the robustness of the pooled effect sizes and identify potential sources of heterogeneity.

Publication bias was assessed through funnel plot analysis, except when fewer than ten studies were included in the quantitative synthesis for a given outcome [23].

## 3. Results

### 3.1. Study Selection

**Figure 1:**
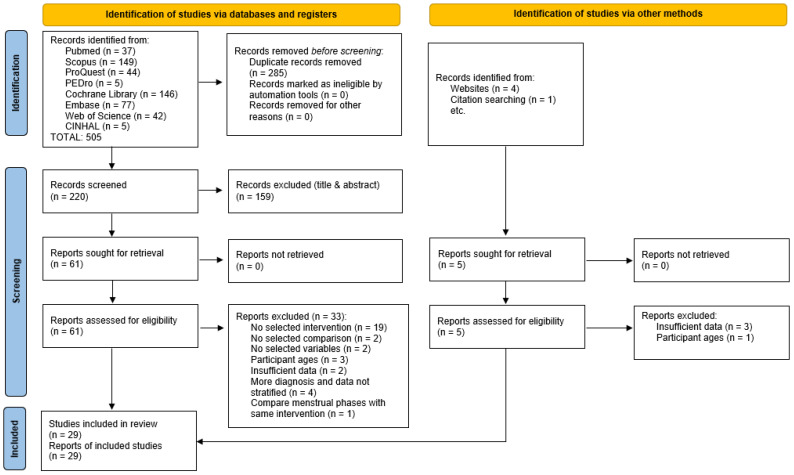
Flow diagram.

The initial database search identified 505 records. After removal of duplicates, 220 articles remained for screening, of which 156 were excluded based on title and abstract review. Following full-text assessment, 29 RCTs were included in the systematic review and meta-analysis (Figure 1). Detailed explanations for exclusions and references are provided in the Supplementary S2.

### 3.2. Characteristics of the Included Studies

A comprehensive overview of the enrolled trials is presented in Table 1. The publication dates spanned from 2011 to 2026, with the research originating across 12 distinct nations: Egypt [24,25,26], Turkey [27,28,29,30,31,32,33,34,35], Iran [36,37,38,39,40,41], Taiwan [42,43], South Korea [44,45], Saudi Arabia [46], India [47,48], Spain [49], Thailand [50], Mexico [20], and Malaysia [51]. A total of 1704 participants were analyzed, with 906 allocated to the experimental group and 798 to the control group. The mean age of the participants ranged between 18 and 32 years. A more detailed description of the characteristics of the participants is provided in Supplementary S3.

All included studies were two-arm RCTs, except for four studies that included three arms [25,32,41,46] and one study that was designed as a four-arm RCT [38]. All studies compared the experimental group with a control group that received no intervention. Regarding the type of TE applied, nine studies implemented aerobic exercise [25,26,27,34,35,41,43,49], seven studies used strength exercise [25,32,36,40,41,44,51], five studies used stretching [32,38,42,46,47], seven studies used mind–body intervention programs [28,29,33,39,45,48,50], and five studies had multimodal programs [20,24,30,31,37].

With respect to intervention parameters, most studies used programs lasting eight weeks; however, in five studies, the duration was four weeks [24,27,34,46], and in nine studies, it was 12 weeks [20,28,29,31,35,43,44,45,50]. One study had a duration of twelve months [42]. The most common weekly frequency was two to three sessions per week, although in five studies the frequency was higher [36,38,39,47,51].

Regarding the intensity parameters of the interventions, very few studies explicitly reported this information objectively. Five studies used maximum heart rate to monitor exercise intensity, ranging from 40–60% [37] to 60–80% [20,25,49], with one study reaching peak workloads of up to 85%. One RCT used 40–60% of heart rate reserve as a reference [35]. The Borg scale was also used by some authors as a tool to monitor perceived exertion, ranging from moderate–hard effort [31,34,49] to hard–very hard [43].

### 3.3. Risk of Bias

**Figure 2:**
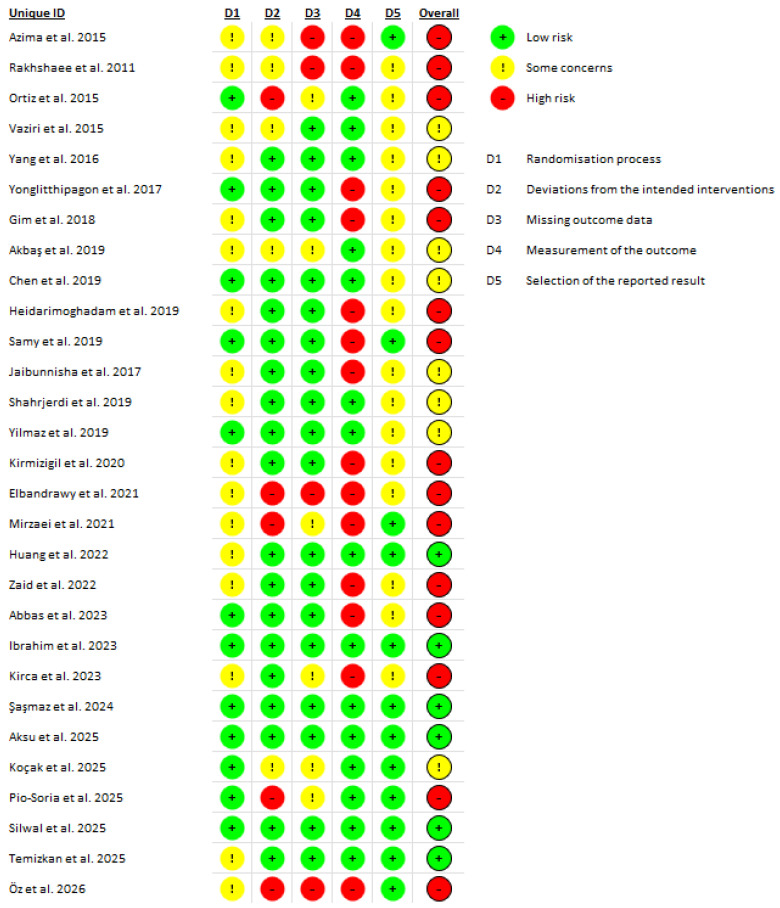
Study-specific risk of bias profiles established according to the investigators’ evaluation (intention-to-treat strategy) [20,24,25,26,27,28,29,30,31,32,33,34,35,36,37,38,39,40,41,42,43,44,45,46,47,48,49,50,51].

Inter-rater reliability for the risk of bias evaluation stood at 77%. Figure 2 graphically displays the detailed risk of bias metrics for the included literature. The most prominent deficiencies contributing to bias risks were associated with the absence of intention-to-treat analysis, missing outcome data, and the lack of blinding of the evaluators.

### 3.4. Quantitative Analysis of the Included Studies

#### 3.4.1. Symptom Severity

**Figure 3:**
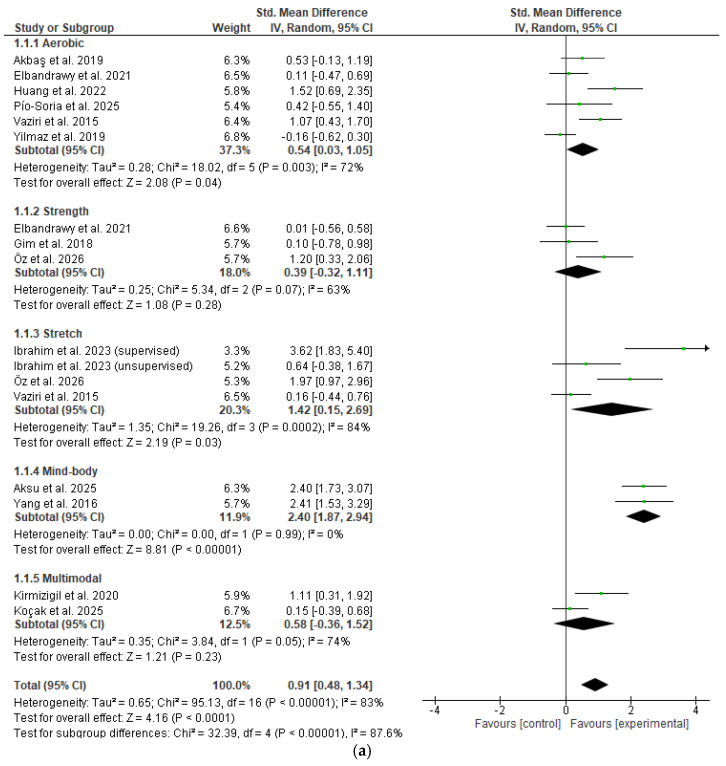

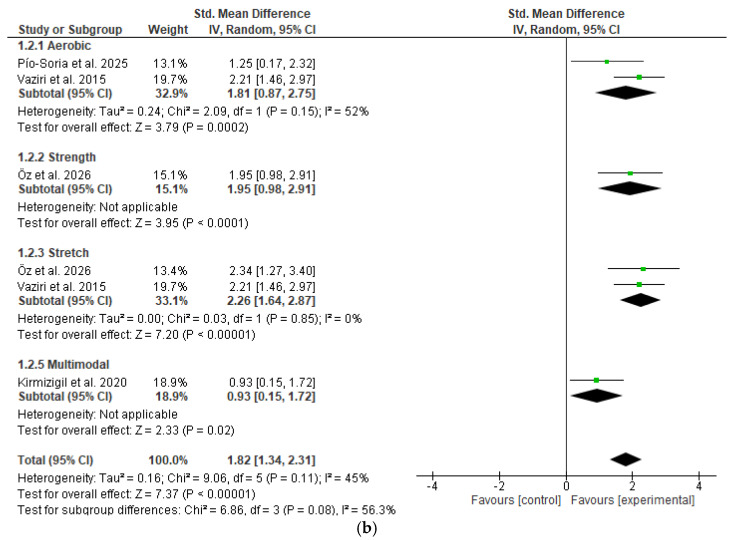
Effect of TE on symptom severity at the (**a**) first and (**b**) second menstrual cycles after intervention [25,27,28,30,31,32,35,41,43,44,45,46,49]. The green dots and black horizontal lines show effect estimates and 95% confidence intervals, respectively.

Thirteen studies [25,27,28,30,31,32,35,41,43,44,45,46,49] evaluated the effect of TE on symptom severity during the first post-intervention menstrual cycle (Figure 3a), using the Menstrual Distress Questionnaire (MDQ) [43,44,45], the Menstrual Symptom Questionnaire (MSQ) [25,28,30,31,32,41], the Premenstrual Syndrome Scale (PMSS) [27,35], or the Verbal Multidimensional Scoring system (VMS) [46]. Overall, TE demonstrated a greater effect compared with control conditions (n = 645; SMD = −0.91; 95% CI: −1.34 to −0.48), although heterogeneity was high (I^2^ = 83%, *p* < 0.05). Subgroup analysis revealed that mind–body interventions (n = 96; SMD = −2.40; 95% CI: −2.94 to −1.87) and stretching interventions (n = 108; SMD = −1.42; 95% CI: −2.69 to −0.15) yielded the largest effect sizes.

Regarding the second post-intervention menstrual cycle, four studies [30,32,41,49] assessed this outcome and reported even greater improvements (n = 197; SMD = −1.82; 95% CI: −2.31 to −1.34). No differences were found between types of TE (Figure 3b).

According to the GRADE approach, the certainty of the evidence for this outcome was very low (downgrade due to risk of bias, inconsistency, and suspected publication bias) (Table 2). Sensitivity analysis showed no change in the direction or size of the effect when studies were sequentially removed (Supplementary S4).

#### 3.4.2. Pain Intensity

**Figure 4:**
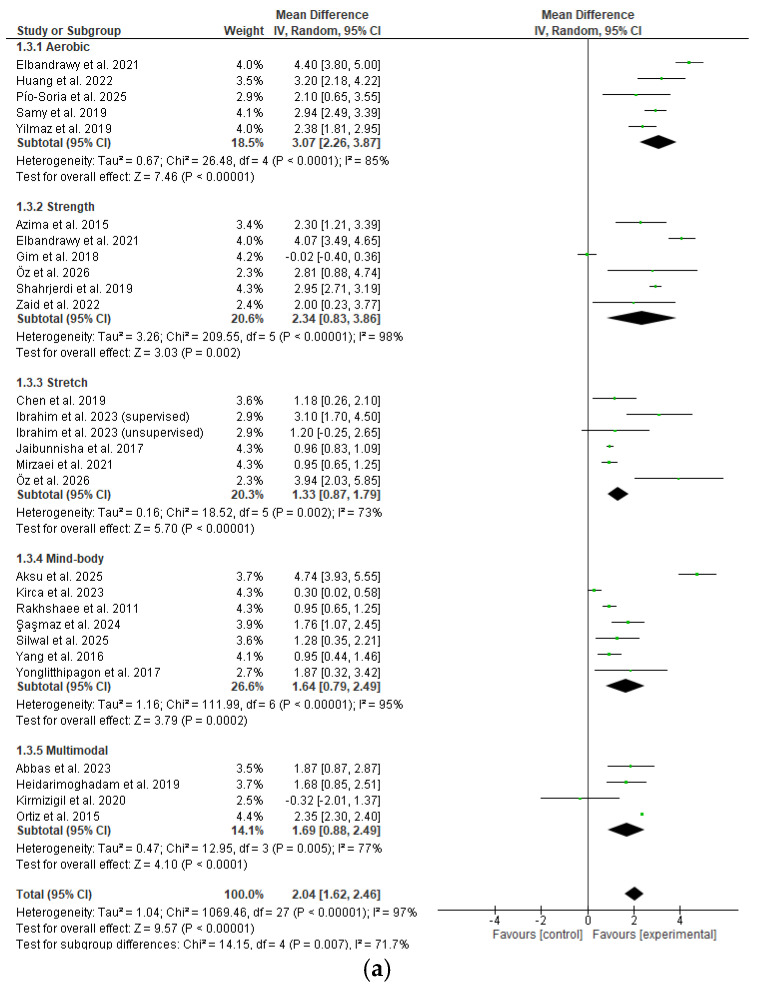

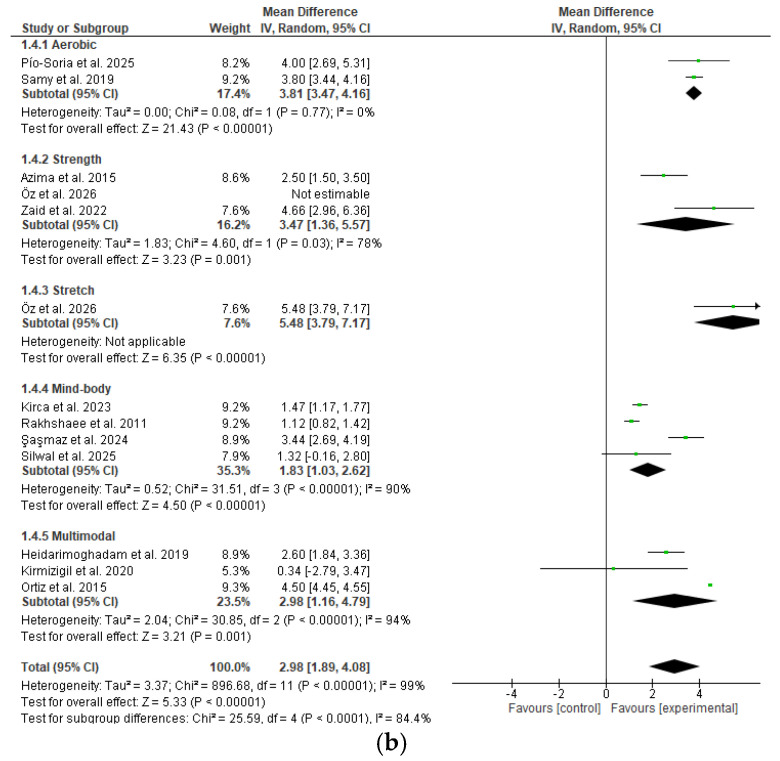
Effect of TE on pain intensity at the (**a**) first and (**b**) second menstrual cycles after intervention [20,24,25,26,28,29,30,32,33,35,36,37,38,39,40,42,43,44,45,46,47,48,49,50]. The green dots and black horizontal lines show effect estimates and 95% confidence intervals, respectively.

Twenty-five studies [20,24,25,26,28,29,30,32,33,35,36,37,38,39,40,42,43,44,45,46,47,48,49,50,51] evaluated pain intensity during the first menstrual cycle following intervention completion (Figure 4a), using either a Visual Analogue Scale (VAS) [20,24,25,26,28,29,30,32,33,36,38,39,40,42,44,45,46,48,49,50,51] or a Numerical Rating Scale (NRS) [47]. Overall, TE demonstrated a larger effect compared with control (n = 1489; MD = −2.04; 95% CI: −2.46 to −1.62), although heterogeneity was considerable (I^2^ = 97%, *p* < 0.05). Subgroup analysis by type of TE showed that aerobic exercise (n = 268; MD = −3.07; 95% CI: −3.87 to −2.26) and strength training (n = 227; MD = −2.34; 95% CI: −3.86 to −0.83) achieved the greatest reductions in pain intensity.

Twelve studies [20,26,29,30,32,33,36,37,39,48,49,51] conducted follow-up assessments during the second menstrual cycle (Figure 4b), reporting further improvements (n = 778; MD = −2.98; 95% CI: −4.08 to −1.89). Aerobic exercise maintained superior effects compared to the other types of exercise (n = 115; MD = −3.81; 95% CI: −4.16 to −3.47).

According to the GRADE approach, the certainty of the evidence for this outcome was very low (downgrade due to risk of bias, inconsistency, and suspected publication bias) (Table 2). Sensitivity analysis showed no change in the direction or size of the effect when studies were sequentially removed (Supplementary S4).

#### 3.4.3. Pain Duration

**Figure 5:**
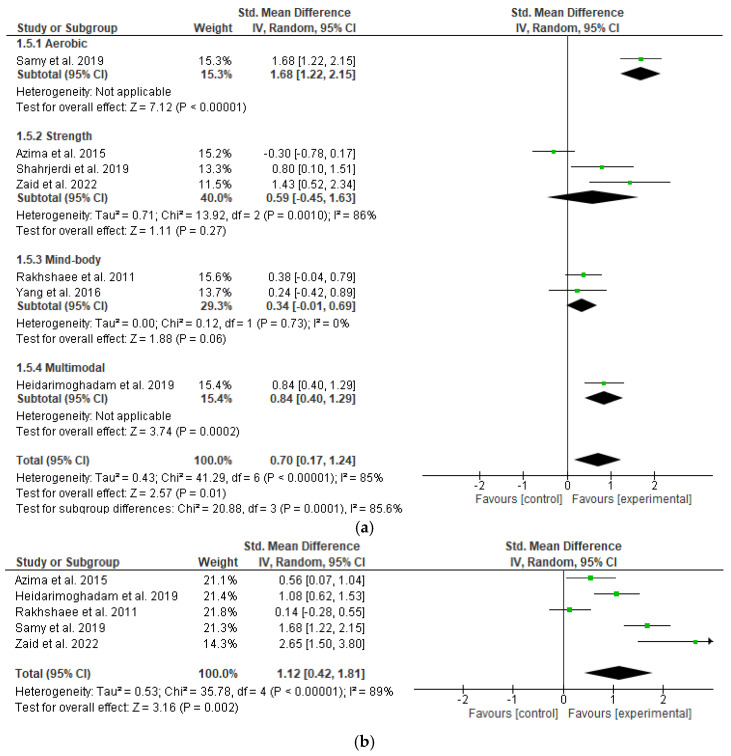
Effect of TE on pain duration at the (**a**) first and (**b**) second menstrual cycles after intervention [26,36,37,39,40,45,51]. The green dots and black horizontal lines show effect estimates and 95% confidence intervals, respectively.

Seven studies [26,36,37,39,40,45,51] assessed the duration of menstrual pain, reported either as hours per day or total hours across the menstrual cycle (Figure 5a). A moderate overall greater effect size was observed in favor of TE compared with the control group (n = 438; SMD = −0.70; 95% CI: −1.24 to −0.17), with substantial heterogeneity (I^2^ = 85%, *p* < 0.05).

The second menstrual cycle following the intervention was evaluated by five studies [26,36,37,39,51] (Figure 5b), showing a greater beneficial effect of TE on reducing menstrual pain duration (n = 368; SMD = −1.12; 95% CI: −1.81 to −0.42).

According to the GRADE approach, the certainty of the evidence for this outcome was low (downgrade due to risk of bias and inconsistency) (Table 2). Sensitivity analysis showed no change in the direction or size of the effect when studies were sequentially removed (Supplementary S4). No change in effect size was observed when studies that measured total hours [39,40], rather than hours per day, were excluded from the analysis.

#### 3.4.4. Secondary Outcomes

**Figure 6:**
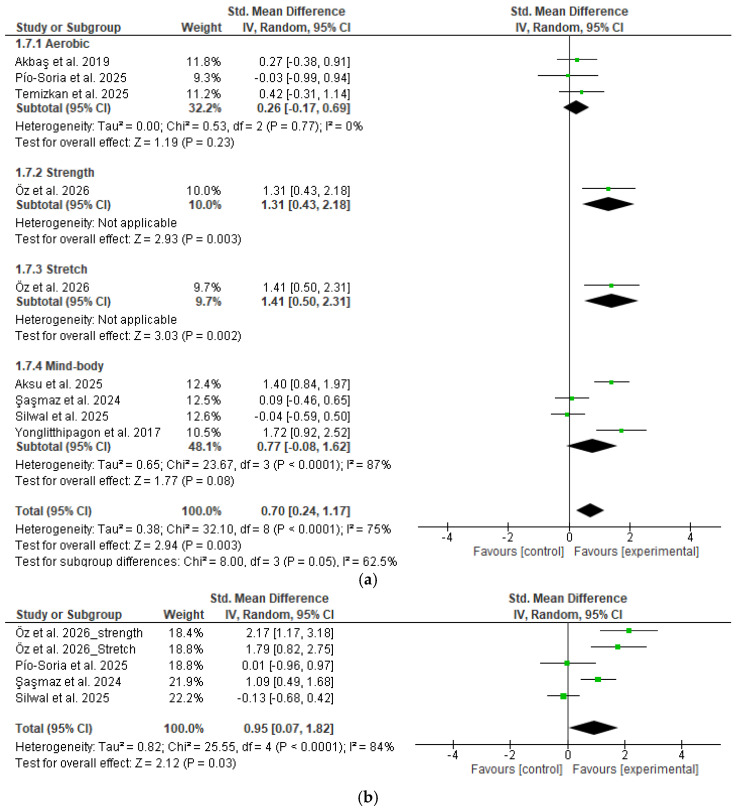
Effect of TE on quality of life at the (**a**) first and (**b**) second menstrual cycles after intervention [27,28,32,33,34,48,49,50]. The green dots and black horizontal lines show effect estimates and 95% confidence intervals, respectively.

Quality of life was assessed in eight RCTs [27,28,32,33,34,48,49,50] during the first menstrual cycle following the intervention using either the 36-Item Short Form Health Survey (SF-36) [27,28,32,33,34,50] or the 12-Item Short Form Health Survey (SF-12) [48,49] (Figure 6a). A moderate overall improvement favoring TE was observed (n = 333; SMD = 0.70; 95% CI: 0.24 to 1.17), although substantial heterogeneity was observed (I^2^ = 75%, *p* < 0.05). Four studies [32,33,48,49] conducted follow-up assessments during the second menstrual cycle (Figure 6b), demonstrating a larger pooled effect (n = 172; SMD = 0.95; 95% CI: 0.07 to 1.82). According to GRADE, the certainty of the evidence was rated as low (downgrade due to risk of bias and inconsistency) (Table 2). Leave-one-out sensitivity analyses indicated that the effect size changed from medium to large when the study by Silwal et al. [48] was excluded (Supplementary S4).

**Figure 7:**
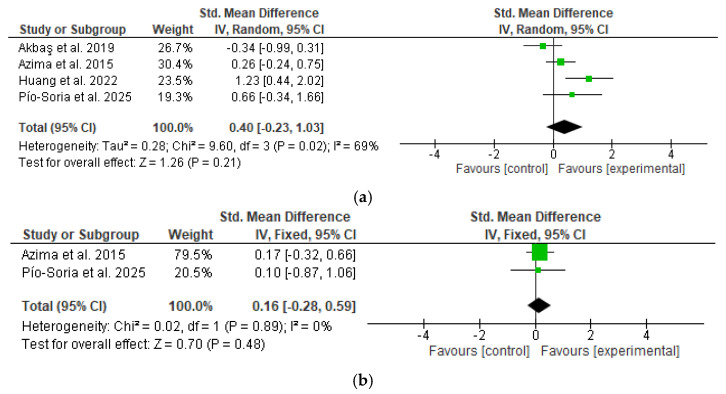
Effect of TE on anxiety at the (**a**) first and (**b**) second menstrual cycles after intervention [27,36,43,49]. The green dots and black horizontal lines show effect estimates and 95% confidence intervals, respectively.

Anxiety was evaluated in four studies [27,36,43,49] during the first menstrual cycle (Figure 7a). Assessment instruments included the Spielberger State–Trait Anxiety Inventory (STAI) [36], the Depression Anxiety Stress Scale-21 (DASS-21) [49], the Beck Depression Inventory (BDI) [27], and a Likert-type scale [43]. TE did not demonstrate superiority over the control condition (n = 148; SMD = 0.40; 95% CI: −0.23 to 1.03). Two studies [36,49] extended follow-up to the second menstrual cycle and reported similar findings (n = 81; SMD = 0.16; 95% CI: −0.28 to 0.59) (Figure 7b). According to GRADE, the certainty of the evidence was rated as very low (downgrade due to risk of bias, inconsistency, and imprecision) (Table 2). The result of the pooled analysis became statistically significant when the study by Akbaş et al. [27] was excluded from the analysis (Supplementary S4).

**Figure 8:**
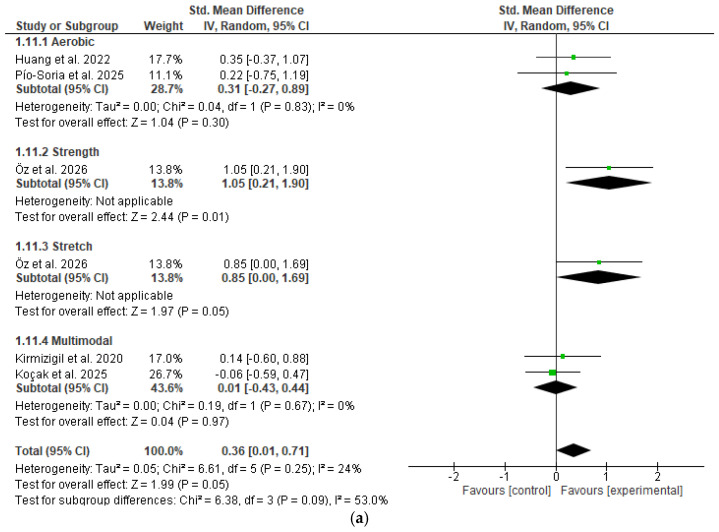

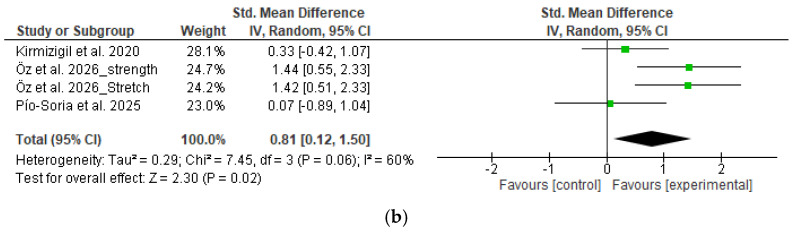
Effect of TE on sleep quality at the (**a**) first and (**b**) second menstrual cycles after intervention [30,31,32,43,49]. The green dots and black horizontal lines show effect estimates and 95% confidence intervals, respectively.

Sleep quality was evaluated in five RCTs [30,31,32,43,49] using the Pittsburgh Sleep Quality Index (PSQI) [30,31,32], a Likert-type scale [43], and the Women’s Health Initiative Insomnia Rating Scale (WHIIRS) [49] (Figure 8a). TE was superior to the control group in improving sleep quality (n = 183, SMD = 0.36; 95% CI: 0.01 to 0.71), with low heterogeneity (I^2^ = 24%, *p* = 0.25). Three studies [30,32,49] conducted follow-up during the second menstrual cycle (Figure 8b), resulting in a greater effect (n = 99, SMD = 0.81; 95% CI: 0.12 to 1.50). According to GRADE, the certainty of the evidence was rated as low (downgrade due to risk of bias and imprecision) (Table 2). In the sensitivity analysis, the overall estimate ceased to be statistically significant when the studies by Huang et al. [43], Öz et al. [32], Pio-Soria et al. [49], and Kirmizigil et al. [30] were removed individually from the model (Supplementary S4).

### 3.5. Secondary Subgroup Analysis

With the aim of identifying potential sources of heterogeneity, subgroup analyses were conducted for the primary outcomes based on the duration of the TE program (≤4 weeks, 5–8 weeks, and ≥9 weeks), type of assessment tool used (MDQ, MSQ, PMSS, VMS, and CVM-22), and risk of bias (low risk, some concerns, and high risk) (Supplementary S5). Subgroup analysis according to assessment tool could not be performed for the pain intensity variable because only one study used the NRS. None of the pooled analyses showed changes in the magnitude of statistical heterogeneity (I^2^).

### 3.6. Dropouts Analysis

Dropout rates were analyzed across all included studies (Supplementary S5). The analysis showed no differences between groups (n = 1507; RR = 0.98; 95% CI: 0.80 to 1.21), with no observed heterogeneity (I^2^ = 0%). Only two studies did not report data on dropout rates [39,41].

### 3.7. Publication Bias

Visual inspection of the funnel plots for symptom severity and pain intensity showed an asymmetric distribution of studies around the pooled effect (Supplementary S6). In both Figures, there is a predominance of positive effects and a limited representation of small studies on the opposite side of the plot. Overall, these findings are compatible with the presence of publication bias.

## 4. Discussion

This systematic review and meta-analysis evaluated the effects of TE on the symptom severity of PD, pain intensity and duration, quality of life, anxiety, and sleep quality in adult women with PD. Our findings suggest that TE may be effective in improving symptom severity of PD and pain intensity and duration, and may be equally beneficial in improving quality of life and sleep quality, but not in reducing anxiety symptoms. However, these findings are supported by very low-to-low-certainty evidence, according to GRADE criteria. Subgroup analysis suggested exploratory trends indicating that mind–body programs and stretching might be associated with improvements in PD symptom severity, while aerobic and strength training appeared to show descriptive tendencies toward reductions in pain intensity. However, these findings stem from indirect cross-study comparisons and must be interpreted with extreme caution, as they may be influenced by confounding factors such as intervention dosage, baseline severity, sample sizes, and different outcome measurement tools.

These findings are consistent with previous meta-analyses [12,13,14]. Unlike recent reviews, the present study incorporated, as a novel aspect, the analysis of key psychosocial variables such as anxiety and sleep quality. Previous research has established an association between the severity of PD and both anxiety and sleep quality, directly influencing pain perception and response to intervention [52,53]. Furthermore, our meta-analysis included only RCTs involving adult women, as PD in adolescents may be influenced by the immaturity of the hypothalamic–pituitary–ovarian axis and the menstrual irregularity characteristic of the early post-menarche years, thereby introducing an additional source of clinical heterogeneity [15]. Moreover, differences in biological maturation, physical activity levels, adherence, and response to exercise between adolescents and adults could act as confounding factors, potentially compromising the internal validity and comparability of the meta-analytic findings. Finally, several RCTs have been conducted since the last published meta-analysis [28,31,32,33,34,48,49], making an updated evidence synthesis necessary.

Our study demonstrates the beneficial effects of TE in reducing both pain intensity and duration in PD. These reductions exceed the values considered as the minimal clinically important difference [54]. Subgroup analyses according to the type of TE suggest that aerobic exercise and strength training may be particularly effective in reducing pain. According to previous studies, the main mechanisms underlying these effects are related to the activation of descending inhibitory pathways, the endogenous release of opioids and endocannabinoids, and anti-inflammatory adaptations [9]. Furthermore, high-intensity aerobic exercise has been shown to increase progesterone levels, which in turn suppress prostaglandin synthesis through an inverse regulatory relationship, thereby producing a considerable reduction in pain intensity [43].

On the other hand, greater reductions in symptom severity were observed with mind–body and stretching interventions. Previous studies have established that the inclusion of elements such as controlled breathing, meditation, and progressive muscle relaxation is key to improving symptom severity, through neuroendocrine modulation of the hypothalamic–pituitary–adrenal axis [55].

The findings of our study indicate that the effects of TE on PD are smaller or may not be fully established within the first menstrual cycle following the intervention, compared with the second cycle, which appears to become more consistent after sustained exposure to TE. The RCTs included in our meta-analysis that reported outcome data during the second post-intervention cycle described a temporal pattern compatible with cumulative benefit, suggesting progressive physiological and neuroplastic adaptations in pain modulation. This temporal pattern is physiologically plausible, as repeated exposure to TE over several weeks has been associated with upregulation of endogenous opioid and serotonergic pathways, as well as modulation of central inhibitory pain circuits (adaptations that are unlikely to stabilize after a single cycle) [12,13]. Taken together, these findings support a dose–time relationship whereby structured exercise maintained for at least two consecutive cycles may progressively enhance endogenous pain-modulatory mechanisms. Nevertheless, high-quality longitudinal trials explicitly modeling inter-cycle changes remain necessary.

Although definitively prescribing an optimal exercise protocol remains challenging due to the low certainty of the current evidence, several actionable clinical trends emerge from our findings. Regarding exercise type, our descriptive data suggest that mind–body and stretching interventions may be preferentially oriented toward reducing overall symptom severity, whereas structured aerobic and strength training appear more suited for targeting pain intensity. Concerning exercise intensity, the analyzed literature intrinsically links structured aerobic and resistance training with moderate-to-high intensities, while mind–body interventions rely on low intensities. A practical prescription based on the findings indicates an optimal protocol of three sessions per week, with interventions lasting 8 to 12 weeks or more to achieve sustained clinical improvements.

The implications of the high risk of bias identified in the included RCTs warrant careful consideration when interpreting the pooled analyses. The most prevalent methodological limitation was the lack of blinding of participants and investigators, an inherent constraint of TE interventions that increases the risk of bias. This factor, together with frequently identified shortcomings in allocation concealment and outcome assessor blinding, introduces a substantial susceptibility to overestimation of treatment effects. Consequently, the presence of these systematic methodological biases reduces the overall certainty of the evidence according to GRADE criteria, placing it at low or very low levels. This lack of methodological robustness in the primary trials limits confidence in the pooled effect sizes for the outcomes analyzed in this meta-analysis.

The high statistical heterogeneity observed across the different pooled analyses in this study limits confidence in the effect estimates. Despite performing subgroup analyses based on intervention duration, clinical assessment tools, and risk of bias, statistical heterogeneity remained high in most of the evaluated models. The only exception was observed in the subgroup of interventions lasting less than 4 weeks (I^2^ = 13%). Therefore, this heterogeneity may be attributed to the marked intrinsic clinical variability of participants across the different RCTs (diagnostic criteria for PD, allowance or restriction of analgesic intake, and baseline pain intensity), as detailed in Supplementary S3.

Regarding study safety and acceptability, our findings suggest that TE is a safe and well-tolerated intervention for patients with PD, as evidenced by attrition rates comparable to those of control groups. The high acceptability of the protocols is further supported by the absence of serious adverse events. Reported side effects were minor and transient, primarily consisting of delayed-onset muscle soreness and sporadic instances of temporary pain exacerbation. These results indicate that, when properly prescribed, exercise does not increase the risk of treatment discontinuation, reinforcing its clinical viability as a low-risk non-pharmacological strategy.

### 4.1. Clinical Implications

The findings of this meta-analysis suggest that TE may represent a useful non-pharmacological option for the management of PD in adult women. The magnitude of the pooled effects observed for symptom severity and pain intensity indicates that structured exercise programs could contribute to meaningful improvements in menstrual-related symptoms and quality of life. However, these potential benefits should be interpreted cautiously given the substantial heterogeneity across studies, the variability in exercise protocols, and the overall low certainty of the evidence. In clinical practice, TE may therefore be considered as a complementary strategy within a multimodal management approach, particularly for women seeking non-pharmacological alternatives or adjuncts to conventional treatments. The observation that larger effects tended to emerge during the second menstrual cycle following the intervention further suggests that sustained engagement in exercise programs may be necessary to achieve clinically relevant benefits. Nevertheless, due to methodological limitations and inconsistency among trials, definitive recommendations regarding the optimal type, intensity, or duration of exercise cannot yet be established.

### 4.2. Limitations

A noteworthy limitation of this systematic review is the marked heterogeneity present among the evaluated endpoints, thereby undermining the overall confidence in the findings. Additionally, subgroup analyses did not meaningfully reduce heterogeneity. Although it was not possible to determine the specific factors underlying this inconsistency, it may be attributable to the wide variability in participants’ demographic and clinical characteristics. Furthermore, the protocols used to deliver TE were highly heterogeneous. Furthermore, the review is constrained by the moderate-to-high risk of bias characterizing multiple included trials. This vulnerability stems principally from a lack of intention-to-treat analyses, data availability limitations, and open-label designs that precluded the blinding of both participants and investigators (with outcome assessors also unblinded in a high proportion of studies). These technical limitations might impact both the precision of the conclusions and their relevance to the general population, and therefore the results should be interpreted with caution. Finally, the exclusion of RCTs including adolescent women may limit the generalizability of the results to this population group.

To address these gaps, subsequent research priorities ought to center on larger, robustly designed randomized trials capable of reducing potential biases and establishing a more definitive foundation of evidence. To achieve an optimal trial design, future studies should focus on the standardization of exercise protocols, incorporate longer follow-up periods exceeding two menstrual cycles to assess sustained effectiveness, and implement better concealment of outcome assessors to ensure objective outcome measurement.

## 5. Conclusions

Our findings demonstrate the therapeutic viability of TE to address the impact of PD in adult women. The low certainty of the evidence and the high risk of bias observed prevent TE from being a substitute for current PD treatments, and it can be considered as a complement to them.

The most consistent benefits were observed in PD symptom severity, pain intensity and duration, quality of life, and, to a lesser extent, sleep quality. The effects were greater during the second menstrual cycle following the intervention. Interventions based on mind–body programs and stretching were associated with a greater reduction in symptom severity, while aerobic and strength exercise had better results on pain intensity.

While our data substantiate the clinical viability of TE, the strength of these pooled estimates remains constrained due to low-to-very-low-certainty ratings derived from the GRADE framework. Consequently, these outcomes must be approached with caution, underscoring the necessity for rigorous, large-scale trials to definitively establish the true efficacy of TE.

## Figures and Tables

**Table 1:** Characteristics of the included studies.

Author/ Year/ Country	Sample and Interventions Characteristics						Intervention Parameters	Outcomes	Drop Outs (EG/CG)
	Experimental Group			Control Group					
	n	Age	Intervention	n	Age	Intervention			
Abbas, M 2023 Egypt [24]	15	24.33 (2.79)	Multimodal	15	24.67 (2.49)	NI	4/w 3 ss/w 30 m/ss	Pain intensity (VAS)	0/0
Akbas, E 2019 Turkey [27]	18	21.10 (1.59)	Aerobic	19	21.20 (1.47)	NI	4 w 3 ss/w 50 m/ss	Symptom severity (PMSS) Pain intensity (VAS)	5/3
Aksu, A 2024 Turkey [28]	30	19.9 (1.2)	Mind–body	30	20.2 (1.2)	NI	12 w 2 ss/w 60 m/ss	Pain intensity (VAS) Symptom severity (MSQ)	0/0
Azima, S 2015 Iran [36]	34	21.08 (1.21)	Strength	34	20.73 (1.08)	NI	8 w 5 ss/w	Pain intensity (VAS) Pain duration (h)	6/6
Chen, HM 2019 Taiwan [42]	63	21.25 (0.47)	Stretch	64	21.26 (0.48)	NI	12 m 3 ss/w 50 m/ss	Pain intensity (VAS)	42/42
Elbandrawy, A 2021 Egypt [25]	35	22.40 (1.94)	Aerobic	35	22.47 (1.55)	NI	8 w 3 ss/w 45 m/ss	Pain intensity (VAS) Symptom severity (MSQ)	0/0/0
	35	22.37 (1.87)	Strength						
Gim, M 2018 South Korea [44]	10	19.20 (1.55)	Strength	10	19.00 (0.82)	NI	12 w 3 ss/w	Pain intensity (VAS) Symptom severity (MDQ)	0/0
Heidarimoghadam, R 2019 Iran [37]	43	18.67 (0.64)	Multimodal	43	18.62 (0.65)	NI	8 w 3 ss/w 20–47 m/ss	Pain intensity (MGPQ) Pain duration (h)	0/0
Huang, WC 2022 Taiwan [43]	15	20.6 (2.5)	Aerobic	15	21.5 (2.1)	NI	10 w 2 ss/w 30–35 m/ss	Pain intensity (VAS) Symptom severity (MDQ) Anxiety (4-point Likert) Sleep quality (4-point Likert)	1/1
Ibrahim, Z 2023 Saudi Arabia [46]	11	21 (1.34)	Stretch (supervised)	11	20.6 (1.29)	NI	4 w 3 ss/w 30–45 m/ss	Pain intensity (VAS) Symptom severity (VMS)	0/0/0
	11	21.2 (1.17)	Stretch (non-supervised)						
Jaibunnisha 2017 India [47]	35	NR	Stretch	35	NR	NI	8 w 6 ss/w 10 m/ss	Pain intensity (NRS)	2/1
Kirca, N 2023 Turkey [29]	30	20.30 (0.46)	Mind–body	30	20.46 (0.50)	NI	12 w 1 ss/w 60 m/ss	Pain intensity (VAS)	2/5
Kirmizigil, B 2020 Turkey [30]	14	22.9 (2.1)	Multimodal	14	23.1 (1.8)	NI	8 w 3 ss/w 45 m/ss	Pain intensity (VAS) Symptom severity (MSQ) Sleep quality (PSQI)	0/0
Koçak, M 2025 Turkey [31]	27	30.3 (6.3)	Multimodal	27	32.7 (6.6)	NI	12 w 3 ss/w 30 m/ss	Symptom severity (MSQ) Sleep quality (PSQI)	5/4
Mirzaei 2021 Iran [38]	20	23.70 (3.46)	Stretch	20	25.15 (3.01)	NI	8 w 4 ss/w	Pain intensity (VAS)	2/2
Ortiz, M 2015 Mexico [20]	83	20.2 (1.8)	Multimodal	77	20.4 (1.2)	NI	12 w 3 ss/w 50 m/ss	Pain intensity (VAS)	13/13
Öz, A 2026 Turkey [32]	19	19.42 (1.46)	Strength	18	20.94 (1.70)	NI	8 w 3 ss/w 50–60 m/ss	Pain intensity (VAS) Symptom severity (MSQ) QoL (SF-36) Sleep quality (PSQI)	3/3
	17	19.24 (1.48)	Stretch						
Pio-Soria, A 2025 Spain [49]	7	20.4 (1.2)	Aerobic	10	20.4 (1.2)	NI	8 w 2 ss/w 26 m/ss	Pain intensity (VAS) Symptom severity (CVM-22) QoL (SF-12) Anxiety (DASS-21) Sleep quality (WHIRS)	3/2
Rakhshaee, Z 2011 Iran [39]	50	20.86 (SD NR)	Mind–body	42	20.45 (SD NR)	NI	8 w 7 ss/w 20 m/ss	Pain intensity (VAS) Pain duration (h)	NR
Samy, A 2019 Egypt [26]	49	21.41 (1.49)	Aerobic	49	21.53 (1.47)	NI	8 w 2 ss/w 60 m/ss	Pain intensity (VAS) Pain duration (h)	0/0
Şaşmaz, Y 2024 Turkey [33]	25	23.9 (2.3)	Mind–body	25	24.2 (2.9)	NI	8 w 2 ss/w 50 m/ss	Pain intensity (VAS) QoL (SF-36)	4/4
Shahrjerdi, S 2019 Iran [40]	17	21.71 (0.99)	Strength	17	22.43 (0.85)	NI	8 w 3 ss/w 45–60 m/ss	Pain intensity (VAS) Pain duration (h)	0/0
Silwal, K 2025 India [48]	26	20.96 (2.59)	Mind–body	25	21.36 (1.99)	NI	8 w	Pain intensity (VAS) QoL (SF-12)	0/1
Temizkan, S 2025 Turkey [34]	15	21.20 (1.01)	Aerobic	15	24 (1.73)	NI	3 w 2 ss/w 45 m/ss	QoL (SF-36)	0/0
Vaziri, F 2015 Iran [41]	35	21.10 (2.07)	Aerobic	35	20.43 (1.83)	NI	8 w 3 ss/w 20 m/ss	Symptom severity (MSQ)	NR
	35	20.81 (1.94)	Stretch						
Yang, NY 2016 South Korea [45]	18	21.0 (SD NR)	Mind–body	18	21.0 (SD NR)	NI	12 w 1 ss/w 60 m/ss	Pain intensity (VAS) Pain duration (h) Symptom severity (MDQ)	0/0
Yilmaz, E 2019 Turkey [35]	35	19.28 (1.63)	Aerobic	36	19.61 (2.11)	NI	12 w 3 ss/w 30 m/ss	Symptom severity (PMSS)	2/1
Yonglitthipagon, P 2017 Thailand [50]	17	19.71 (1.36)	Mind–body	17	20.06 (1.03)	NI	12 w 2 ss/w 30 m/ss	Pain intensity (VAS) QoL (SF-36)	0/0
Zaid, N 2022 Malaysia [51]	12	22.58 (0.79)	Strength	12	22.58 (0.90)	NI	8 w 5 ss/w 10 m/ss	Pain intensity (VAS) Pain duration (h)	0/0

Abbreviations: n = sample size; EG = experimental group; NI = no intervention; CG = control group; w = weeks; ss = sessions; m = minutes; VAS = Visual analogue Scale; PMSS = Premenstrual Symptom Scale; MSQ; Menstrual Symptom Questionnaire; h = hours; MDQ = Menstrual Distress Questionnaire; MGPQ = McGill Pain Questionnaire; VMS = Verbal Multidimensional Scoring; NRS = Numerical Rating Scale; SF-12 = 12-item Short Form Health Survey; WHIIRS = Women’s Health Initiative Insomnia Rating Scale; PSQI = Pittsburgh Sleep Quality Index; SF-36 = 36-item Short Form Health Survey; CVM-22 = Menstrual Quality of Life Questionnaire; DASS-21 = Depression, Anxiety and Stress Scale—21 items.

**Table 2:** Grading of evidence certainty based on the reviewers’ final evaluation.

Certainty Assessment							Nº of Patients		Effect	Certainty	Importance
№ of Studies	Study Design	Risk of Bias	Inconsistency	Indirectness	Imprecision	Other Considerations	TE	Control	Absolute (95% CI)		
Symptom severity (assessed with: MDQ, MSQ, PMSS, VMS)											
13	RCT	serious ^a^	serious ^b^	not serious	not serious	publication bias strongly suspected ^c^	370	275	SMD, 0.91 (0.48 to 1.34)	⨁◯◯◯ Very low ^a,b,c^	CRITICAL
Pain intensity (assessed with: VAS, NRS)											
25	RCT	serious ^a^	serious ^b^	not serious	not serious	publication bias strongly suspected ^c^	782	707	MD, −2.04 (−2.46 to −1.62)	⨁◯◯◯ Very low ^a,b,c^	CRITICAL
Pain duration (assessed with: hours per day; hours per menstrual cycle)											
7	RCT	serious ^a^	serious ^b^	not serious	not serious	none	223	215	SMD, −0.70 (−1.24 to −0.17)	⨁⨁◯◯ Low ^a,b^	CRITICAL
Quality of life (assessed with: SF-36, SF-12)											
8	RCT	serious ^a^	serious ^b^	not serious	not serious	none	174	159	SMD, 0.70 (0.24 to 1.17)	⨁⨁◯◯ Low ^a,b^	IMPORTANT
Anxiety (assessed with: STAI, DASS-21, BDI, Likert-type scale)											
4	RCT	serious ^a^	serious ^b^	not serious	serious ^d^	none	72	76	SMD, 0.40 (−0.23 to 1.03)	⨁◯◯◯ Very low ^a,b,d^	IMPORTANT
Sleep quality (assessed with: PSQI, WHIIRS)											
5	RCT	serious ^a^	not serious	not serious	serious ^d^	none	99	84	SMD, 0.33 (0.02 to 0.63)	⨁⨁◯◯ Low ^a,d^	IMPORTANT

Abbreviations: TE: therapeutic exercise; CI: confidence interval; SMD: standardized mean difference. ^a^ According to the ROB2 assessment, the included studies exhibited some concerns and a high risk of bias. ^b^ Substantial heterogeneity across studies. ^c^ Suspected publication bias based on visual asymmetry of the funnel plot. ^d^ Low statistical power and uncertainty in the result.

## Data Availability

The data presented in this study are available on request from the corresponding author. These data were extracted and analyzed from published studies.

## Supplementary Materials

The following supporting information can be downloaded at: https://www.mdpi.com/article/10.3390/jcm15124418/s1; Supplementary S1: PRISMA checklist and complete search strategy; Supplementary S2: studies excluded, with reasons; Supplementary S3: detailed description of the characteristics of the participants; Supplementary S4: sensitivity analysis; Supplementary S5: subgroup analysis; Supplementary S6: funnel plots.

## Abbreviations

The following abbreviations are used in this manuscript:

PD	Primary dysmenorrhea
TE	Therapeutic exercise
PRISMA	Preferred Reporting Items for Systematic Reviews and Meta-Analyses
CENTRAL	Cochrane Central Register of Controlled Trials
RCT	Randomized controlled trial
MeSH	Medical Subject Headings
COMET	Core outcome measures in effectiveness trials
RoB	Risk of bias
GRADE	Grading of Recommendations Assessment, Development and Evaluation
MD	Mean difference
SMD	Standardized mean difference
CI	Confidence interval
MDQ	Menstrual Distress Questionnaire
MSQ	Menstrual Symptom Questionnaire
PMSS	Premenstrual Syndrome Scale
VMS	Verbal Multidimensional Scoring system
VAS	Visual Analogue Scale
NRS	Numerical Rating Scale
SF-36	36-Item Short Form Health Survey
SF-12	12-Item Short Form Health Survey
STAI	Spielberger State–Trait Anxiety Inventory
DASS-21	Depression Anxiety Stress Scale-21
BDI	Beck Depression Inventory
PSQI	Pittsburgh Sleep Quality Index
WHIIRS	Women’s Health Initiative Insomnia Rating Scale
