# Supplementary material for: Effectiveness of Therapeutic Exercise in Reducing the Severity of Primary Dysmenorrhea and Associated Symptoms: A Systematic Review and Meta-Analysis
Source: J Clin Med. 2026 Jun 7;15(12):4418. doi: 10.3390/jcm15124418 (PMC13301083; doi:10.3390/jcm15124418)
Supplement: Supplementary file 1 [file jcm-15-04418-s001.zip › Supplementary S2.pdf]

Supplementary Appendix S2_ Detailed explanations for exclusions and references

Abdelrahman, Asmaa Y., Soheir M. El-Kosery, Amr H. Abbassy, y Afaf M. Botla. 2024. «Effect of Aquatic Exercise versus Aerobic Exercise on Primary Dysmenorrhea and Quality of Life in Adolescent Females: A Randomized Controlled Trial». *Physiotherapy Research International: The Journal for Researchers and Clinicians in Physical Therapy* 29 (3): e2095. https://doi.org/10.1002/pri.2095.

Reason: No selected intervention.

Ahsan, Adeeba, Pramod Sahu, Jyoti Sharma, Aksh Chahal, y Mohammad Sidiq. 2025. «Comparison of Core and Pelvic Floor Strengthening versus Yoga Poses for Primary Dysmenorrhea among Young Adult Females: A Comparative Experimental Study». Journal od Clinical and Diagnostic research 19: YC01-6. https://doi.org/10.7860/JCDR/2025/79059.21422.

Reason: No selected intervention.

Alkhatib, Asem, Hoda Alshikh Ahmad, Ci Zhang, Wenwen Peng, y Xianhong Li. 2024. «Impact of Traditional Chinese Baduanjin Exercise on Menstrual Health among International Female Students Studying in China: A Randomized Controlled Trial». *Frontiers in Public Health* 12: 1259634. https://doi.org/10.3389/fpubh.2024.1259634.

Reason: More diagnosis and data not stratified.

Beaumont, Tara, Kate Phillips, M. Louise Hull, y Rose Green. 2022. «Does Group Physiotherapy Improve Pain Scores and Reduce the Impact of Pelvic Pain for Women Referred with Persistent Pelvic Pain? A Clinical Trial». *Journal of Endometriosis and Pelvic Pain Disorders* 14 (4): 169-77. https://doi.org/10.1177/22840265221141527.

Reason: More diagnosis and data not stratified.

Behbahani, Bahar Morshed, Lala Ansaripour, Marzieh Akbarzadeh, Najaf Zare, y Mohammad Javad Hadianfard. 2016. «Comparison of the Effects of Acupressure and Self-Care Behaviors Training on the Intensity of Primary Dysmenorrhea Based on McGill Pain Questionnaire among Shiraz University Students». *Journal of Research in Medical Sciences: The Official Journal of Isfahan University of Medical Sciences* 21: 104. https://doi.org/10.4103/1735-1995.193176.

Reason: No selected intervention.

Boztaş Elverişli, Gizem, Nurcan Armağan, y Esra Atilgan. 2023. «Comparison of the Efficacy of Pharmacological and Nonpharmacological Treatments in

Women with Primary Dysmenorrhea: Randomized Controlled Parallel-Group Study». *Ginekologia Polska* 94 (9): 687-97. https://doi.org/10.5603/GP.a2022.0009.

Reason: No selected intervention.

Çelik, Aslı Sis, y Serap Ejder Apay. 2021. «Effect of Progressive Relaxation Exercises on Primary Dysmenorrhea in Turkish Students: A Randomized Prospective Controlled Trial». *Complementary Therapies in Clinical Practice* 42: 101280. https://doi.org/10.1016/j.ctcp.2020.101280.

Reason: No selected intervention.

Chaudhuri, Aditi, Amarjeet Singh, y Lakhbir Dhaliwal. 2013. «A Randomised Controlled Trial of Exercise and Hot Water Bottle in the Management of Dysmenorrhoea in School Girls of Chandigarh, India». *Indian Journal of Physiology and Pharmacology* 57 (2): 114-22.

Reason: Participant ages.

Çinar, Gamze Nalan, Sezcan Mümüşoğlu, Türkan Akbayrak, Ceren Gürşen, y Serap Özgül. 2026. «A Comparison of the Effects of Aerobic Exercise and Yoga Training in Primary Dysmenorrhea: A Single-Blind Randomized Clinical Trial». *European Journal of Obstetrics, Gynecology, and Reproductive Biology* 317: 114853. https://doi.org/10.1016/j.ejogrb.2025.114853.

Reason: No selected intervention.

Dehnavi, Zahra Mohebbi, Farzaneh Jafarnejad, y Zahra Kamali. 2018. «The Effect of aerobic exercise on primary dysmenorrhea: A clinical trial study». *Journal of Education and Health Promotion* 7: 3. https://doi.org/10.4103/jehp.jehp_79_17.

Reason: Insufficient data.

Erdoğan, Meryem, Şenay Ünsal Atan, y İrem Şenyuva. 2024. «The Effect of Yoga on Premenstrual Distress, Quality of Life, and Stress Level». *Holistic Nursing Practice* 38 (6): 361-70. https://doi.org/10.1097/HNP.0000000000000661.

Reason: More diagnosis and data not stratified.

Erol, Büşra Nur, Ceren Gürşen, Sezcan Mümüşoğlu, y Serap Özgül. 2025. «Pain Neuroscience Education Versus Biomedical Pain Education with Exercise in Primary Dysmenorrhea: A Randomized Controlled Trial». *Healthcare* (Basel, Switzerland) 13 (16): 1954. https://doi.org/10.3390/healthcare13161954.

Reason: No selected intervention.

Fallah, F., y M. Mirfeizi. 2018. «How is the quality and quantity of primary dysmenorrhea affected by physical exercises? A study among Iranian students | Cochrane Library». *Int J Women's Health Reprod Sci* 6: 60-66.

Reason: Participant ages.

Fu, Jiali, Xinxin Tan, Yao Li, et al. 2023. «Efficacy of Tuina plus Ba Duan Jin for Primary Dysmenorrhea Due to Cold-Induced Blood Stasis». *Journal of Acupuncture and Tuina Science* 21 (6): 470-75. https://doi.org/10.1007/s11726-023-1406-0.

Reason: No selected intervention.

Gurpreet, K., K. Jaspreet, y Kaur Ravneet. 2018. «The Effect of Exercises in Primary Dysmenorrhea among Young Females of Adesh University». *Indian Journal of Physiotherapy and Occupational Therapy—An International Journal*. https://www.semanticscholar.org/paper/The-Effect-of-Exercises-in-Primary-Dysmenorrhea-of-Gurpreet-Jaspreet/d97f268c44bb86416e2cf561c270311aa29bbfa3.

Reason: No selected intervention.

Hansen, K. E., B. Brandsborg, U. S. Kesmodel, et al. 2023. «Psychological Interventions Improve Quality of Life despite Persistent Pain in Endometriosis: Results of a 3-Armed Randomized Controlled Trial». *Quality of Life Research: An International Journal of Quality of Life Aspects of Treatment, Care and Rehabilitation* 32 (6): 1727-44. https://doi.org/10.1007/s11136-023-03346-9.

Reason: No selected intervention.

Huang, Wen-Ching, Pei Chi Chiu, y Chi Hong Ho. 2022. «The Sprint-Interval Exercise Using a Spinning Bike Improves Physical Fitness and Ameliorates Primary Dysmenorrhea Symptoms Through Hormone and Inflammation Modulations: A Randomized Controlled Trial». *Journal of Sports Science & Medicine* 21 (4): 595-607. https://doi.org/10.52082/jssm.2022.595.

Reason: No selected intervention.

Imtiaz, Iqra, y Huma Riaz. 2022. «Effects of High Intensity Aerobic Training on Symptomatology of Primary Dysmenorrhoea». *JPMA. The Journal of the Pakistan Medical Association* 72 (12): 2515-18. https://doi.org/10.47391/JPMA.5158.

Reason: No selected intervention.

Kachapeswaran, Geetha, y R. Elangovan. 2022. «Effect of yogic practices on selected risk factors among adolescent girls suffering with dysmenorrhea». *Asian Journal of Microbiology, Biotechnology and Environmental Sciences*, 401-7. https://doi.org/10.53550/AJMBES.2022.v24i02.033.

Reason: No selected variables.

Kannan, Priya, Cathy M. Chapple, Dawn Miller, Leica Claydon-Mueller, y G. David Baxter. 2019. «Effectiveness of a Treadmill-Based Aerobic Exercise Intervention on Pain, Daily Functioning, and Quality of Life in Women with Primary Dysmenorrhea: A Randomized Controlled Trial». *Contemporary Clinical Trials* 81: 80-86. https://doi.org/10.1016/j.cct.2019.05.004.

Reason: Insufficient data.

Koçoğlu, Selda, Ömer Şevgin, y Beyzanur Dikmen Hoşbaş. 2025. «Comparison of Motor Imagery Focused Pelvic Floor Exercises and Relaxation Exercises for Treating Dysmenorrhea: A Randomized Controlled Study». *Taiwanese Journal of Obstetrics & Gynecology* 64 (4): 671-77. https://doi.org/10.1016/j.tjog.2024.12.029.

Reason: No selected intervention.

Kovács, Zoltán, Gabriella Hegyi, y Henrik Szőke. 2023. «The Effect of Exercise on Pulsatility Index of Uterine Arteries and Pain in Primary Dysmenorrhea». *Journal of Clinical Medicine* 12 (22): 7021. https://doi.org/10.3390/jcm12227021.

Reason: No selected variables.

M, Sandhiya, Senthil Selvam P, Manoj Abraham M, et al. 2020. «A Study To Compare The Effects Of Aerobic Exercise Versus Core Strengthening Exercise Among College Girls With Primary Dysmenorrhea». *International Journal of Research in Pharmaceutical Sciences* 11 (4): 2692-97. https://doi.org/10.26452/ijrps.v11iSPL4.4542.

Reason: Participant ages.

Marques Abreu da Fonseca, Juliany, Caroline Santos Radmann, Fabiana Teixeira de Carvalho, y Laiana S. de Andrade Mesquita. 2016. «The Influence of the Pilates Method on Muscular Flexibility, Symptoms, and Quality of Life in Women with Primary Dysmenorrhea». Scientia Medica. *Scientia Medica* 26 (2): 6.

Reason: No selected comparison.

Monika, null, Uma Singh, Archana Ghildiyal, Sarswati Kala, y Neena Srivastava. 2012. «Effect of Yoga Nidra on Physiological Variables in Patients of Menstrual Disturbances of Reproductive Age Group». *Indian Journal of Physiology and Pharmacology* 56 (2): 161-67.

Reason: More diagnosis and data not stratified.

Nahas, Engy M. El, Doaa Tammam Atia, Mai H. Hassan, Ahmed Abd El-Moneim Abd El-Hakim, y Sally Mohamed Sae'd Mahmoud. 2024. «Effect of Pilates Exercise versus Yoga on Primary Dysmenorrhea in Adolescent Girls». *SPORT*

TK-EuroAmerican Journal of Sport Sciences, 5-5. https://doi.org/10.6018/sportk.605701.

Reason: No selected intervention.

Pazoki, Hassan, Golbano Bolouri, Farah Farokhi, y Mohammad Ali Azerbayjani. 2016. «Comparing the effects of aerobic exercise and *Foeniculum vulgare* on pre-menstrual syndrome». *Middle East Fertility Society Journal* 21 (1): 61-64. https://doi.org/10.1016/j.mefs.2015.08.002.

Reason: No selected comparison.

Shetty, Deeksha P., Neetinakumar J. Patil, G. Shyamala, et al. 2025. «Yoga as a holistic intervention for primary dysmenorrhea: A pilot study on pain, mental well-being, and quality of life». *Advances in Integrative Medicine* 12 (4). https://doi.org/10.1016/j.aimed.2025.100558.

Reason: No selected intervention.

Soni, Pooja, y Devangi Desai. 2021. «Effectiveness of Pilates and Self-Stretching Exercise on Pain and Quality of Life in Primary Dysmenorrhea" - A Comparative Study». *Indian Journal of Physiotherapy and Occupational Therapy - An International Journal* 15 (3): 129-38. https://doi.org/10.37506/ijpot.v15i3.16173.

Reason: No selected intervention.

Yadav, Sudha, Shabnam Joshi, y Sonu Punia. 2024. «Efficacy of Combined Exercise Training during Different Menstrual Phases in Young Students with Primary Dysmenorrhoea». *Physiotherapy Research International: The Journal for Researchers and Clinicians in Physical Therapy* 29 (4): e2131. https://doi.org/10.1002/pri.2131.

Reason: Compare menstrual phases with same intervention.

Yang, Min-Yi, Hao-Yu Chen, Chi-Hong Ho, y Wen-Ching Huang. 2025. «Impact of Probiotic Supplementation and High-Intensity Interval Training on Primary Dysmenorrhea: A Double-Blind, Randomized Controlled Trial Investigating Inflammation and Hormonal Modulation». *Nutrients* 17 (4): 622. https://doi.org/10.3390/nu17040622.

Reason: No selected intervention.

Yildirim, Muhammed Şeref, Merve Çoğ, Büşra Mehder Akbaş, Sinem Salar, y Hilal Keklicek. 2025. «Acute Effects of Virtual Reality-Based Relaxation and Exergaming on Primary Dysmenorrhea Symptoms». *Turkish Journal of Medical Sciences* 55 (2): 377-85. https://doi.org/10.55730/1300-0144.5981.

Reason: No selected intervention.

Yosri, Mahitab Mohamed, Hamada Ahmed Hamada, Marwa Abd El-Rahman Mohamed, y Amel Mohamed Yousef. 2022. «Effect of Different Squatting Exercises on Menstrual Aspects, Pelvic Mechanics and Uterine Circulation in Primary Dysmenorrhoea: A Randomised Controlled Trial». *Journal of Obstetrics and Gynaecology: The Journal of the Institute of Obstetrics and Gynaecology* 42 (8): 3658-65. https://doi.org/10.1080/01443615.2022.2153021.

Reason: No selected intervention.
