# Supplementary material for: Effectiveness of Therapeutic Exercise in Reducing the Severity of Primary Dysmenorrhea and Associated Symptoms: A Systematic Review and Meta-Analysis
Source: J Clin Med. 2026 Jun 7;15(12):4418. doi: 10.3390/jcm15124418 (PMC13301083; doi:10.3390/jcm15124418)
Supplement: Supplementary file 1 [file jcm-15-04418-s001.zip › Supplementary S3.pdf]

Supplementary Appendix S3_ Detailed description of the characteristics of the participants

Study / Author (Year)	Diagnostic Criteria for Primary Dysmenorrhea	Exclusion of Secondary Dysmenorrhea / Endometriosis / Adenomyosis / Other Pelvic Pathology	Parity	Use of NSAIDs or Other Analgesics	Use of Hormonal Contraceptives	Menstrual Cycle Regularity	Pain Intensity / Baseline Symptom Severity	Baseline Physical Activity Level
Abbas (2023)	Based on the menstrual history and clinical examination. Sonography was used to confirm	The study did not include women genital organs diseases, abnormal vaginal bleeding	Not reported	Excluded undergoing pharmacological or non-pharmacological methods for pain relief	Excluded oral contraceptive users	Excluded infrequent or irregular menstrual cycles	Severe primary dysmenorrhea by VAS	Any history of regular exercises was excluded
Akbaş (2019)	Self-reported dysmenorrhea	Excluded known genital and chronic diseases	Not reported	Not reported	Excluded taking oral contraceptive pills during the study	Regular menstrual cycles (21-35 days)	Menstrual pain scoring at least 4/10 VAS for previous 2 consecutive months	Not reported
Aksu (2025)	A gynecologist examined for PD diagnosis and screening for pelvic or gynecological diseases.	Excluded diagnosis of pelvic or gynecologic diseases or secondary dysmenorrhea	Excluded having a previous pregnancy experience	Not reported	Excluded use of intrauterine devices and contraceptives	Regular menstrual periods (21–35-day intervals and lasting 3-8 days)	Pain score above 4/10 on VAS, and Menstruation Symptom Scale score of 60 and above	Excluded practicing yoga regularly
Azima (2015)	Self-reported dysmenorrhea	Not suffering from systemic diseases or diseases in the genital organs	Not reported	Not using pharmacological methods for pain	Not reported	Not reported	Pain intensity of 5/10 or above in VAS	Not reported

Chen (2019)	According to the primary dysmenorrhea consensus guideline	No prior history of gynecological disease or secondary dysmenorrhea	Not reported	No analgesics medication taken during the study period	Not reported	Not reported	Average experience of in the last 6 months superior to 4/10 points on the VASP.	Not reported
Elbandrawy (2021)	Diagnosis according to the guidelines of the Society of Obstetricians and Gynaecologists of Canada	No pelvic pathology	Not reported	Non-sedating medications, vitamin supplements, or mineral supplements during the three menstrual cycles prior to the trial	Not reported	Regular menstrual cycle (28-32 days)	Menstruation cramps assessed as at least 3-4 (moderate to severe) by Menstrual Symptom Questionnaire	Not being a professional athlete
Gim (2018)	Self-reported dysmenorrhea	Excluded pelvic inflammatory disease, endometriosis	Have never given birth	Refrain from using pain killers, heat therapy during the study	Excluded oral contraceptives	Regular for at least 3 months and has a cycle of 25-35 days	Measurement of menstrual pain above 4/10 in VAS	No previous experience with gym ball exercises
Heidarimoghadam (2019)	Mild to moderate dysmenorrhea during the last three periods according to the McGill Pain Questionnaire	Not reported pelvic pathology	Not reported	No use of any particular chemical or herbal drug affecting dysmenorrhea during the study	Not reported	Regular cycles of every 21 to 35 d	Mild to moderate dysmenorrhea according to the McGill pain scale (1 < score <6.6).	Lack of regular trained exercises
Huang (2022)	Self-reported dysmenorrhea	Excluded menstrual disorders	Not reported	Allowed the use of analgesics	Not reported	Not reported	VAS > 5/10 for dysmenorrhea	Not reported

Ibrahim (2023)	Self-reported dysmenorrhea	Excluded secondary dysmenorrhea	Being not married	Not reported	Excluded taking any hormonal medications that might interfere	Regular menstrual cycle length of 24-35 days, cycle bleed lasting for 3-7 days	Pain level of 4 or more in the initial VAS	Moderate physical activity (less than 3 days per week of physical activities)
Jaibunnisha (2017)	Self-reported dysmenorrhea	Excluded history of any systematic diseases, traumatic injury, any other gynaecological diseases	Not reported	Not reported	Not reported	Regular menstrual cycle	Not reported	Not reported
Kirca (2023)	Self-reported dysmenorrhea	Excluded secondary dysmenorrhea	Having no childbearing	Not reported	Non-use of hormonal contraceptives	Regular menstruation (for the last six months)	VAS value of 6/10 and over	Not reported
Kirmizigil (2020)	Self-reported dysmenorrhea	Excluded secondary dysmenorrhea	Not having a history of a previous abortion or giving birth	Instructed not to use any pain-relieving method, medication	Excluded intrauterine devices	Regular menstrual cycles (one every 24-35 days)	Severity of at least 5 on the Visual Analog Scale	Not reported
Koçak (2025)	Self-reported dysmenorrhea	Excluded diagnosed gynecological disease	Not reported	Excluded receiving pharmacologic treatment for menstrual symptoms	Not reported	Menstrual cycle between 21 and 35 days on average	Not reported	Excluded exercising regularly

Mirzaei (2021)	Self-reported dysmenorrhea	Excluded abnormal uterine or pelvic ultrasound, history of surgery, especially in the abdominal and pelvic area, and other systemic, metabolic, or cardiovascular diseases	Having no history of pregnancy	Excluded the use of any type of medication, specifically use of painkillers	Excluded use of contraceptive pills and hormone therapy	Regular menstrual cycles (21 to 35 days and duration of 3 to 7 days)	Pai intensity ranging from 4/10 to 10/10 on a VAS.	No physical activity and no chronic sports activities
Ortiz (2015)	Painful menstruation in the last four previous months	Excluded secondary dysmenorrhea	Not reported	Excluded regular use of non-steroidal anti-inflammatory drugs or drugs indicated for dysmenorrhea	Not reported	Not reported	Pain intensity greater than 4/10 mm on a VAS	Sedentary lifestyle (less than 25 min/day in physical activity three times a week)
Öz (2026)	Self-reported dysmenorrhea	Excluded conditions causing pelvic pathology/secondary dysmenorrhea	Nulliparous	Not reported	Excluded hormonal contraceptives	Regular menstrual cycle between 24 and 35 days	Score of 60 points or above on the menstrual symptoms scale	Did not have regular exercise routines
Pio-Soria (2025)	Clinical diagnosis of primary dysmenorrhea	Absence of signs typically associated with secondary dysmenorrhea	Not reported	Not reported	Excluded use of contraceptive pills in the past 3 months	Regular menstrual cycle (24-38 days)	Pain score ≥ 4/10 on the VAS	<3 h/week of moderate-intensity physical activity
Rakhshaee (2011)	Cramping pain in the lower abdomen occurring at the onset of menstruation	No medical history of other gynecological diseases	Not reported	Absence of hormonal treatment and nonpharmacologic methods before menstruation	Absence of hormonal treatment	Having regular menstrual cycles	Moderate to severe pain intensity on the VAS	Not reported

Samy (2019)	Self-reported dysmenorrhea	Excluded secondary dysmenorrhea and known genitourinary system diseases	Not reported	Not reported	Excluded contraceptive methods	Regular menstrual cycles (30-35 days) and menstrual bleeding of 3-10 days	Menstrual pain score over 4/10 on VAS	Not reported
Şaşmaz (2024)	Self-reported dysmenorrhea	Excluded history of secondary dysmenorrhea	Excluded history of birth or current pregnancy	Not reported	Excluded oral contraceptives at least 6 months before	Regular menstrual cycle (28±7 days)	VAS score higher than 4/10in the last 6 months	Not reported
Shahrjerdi (2019)	Primary dysmenorrhea of more than 6 months	Excluded polycystic ovary disease, and pelvic infection or pelvic pain	Lacked a history of pregnancy	Allowed to take only NSAIDs as medication	Not reported	Excluded developing irregular menstrual cycle	Reported pain between 5/10 and 10/10 on VAS	Excluded attending regular exercise training in the last 6 months
Silwal (2025)	Diagnosed based on primary dysmenorrhea consensus guidelines	Excluded gynecological or psychiatric diseases, as well as those with secondary dysmenorrhea	Not reported	Not reported	Excluded using oral contraceptive pills or undergoing hormonal therapies within the past three months	Regular menstrual cycle lasting between 28 ± 7 days	Menstrual pain severity rated as 3/10 or more on a VAS	Not reported
Temizkan (2025)	Diagnosed with primary dysmenorrhea by a gynecologist	Excluded having endometriosis, adenomyosis, myoma uteri, and endometrial polyp	Not reported	Not reported	Excluded using an intrauterine device	Not reported	Menstrual pain of 5/10 or more on VAS	Not reported

Vaziri (2015)	Self-reported dysmenorrhea	Excluded pelvic diseases such as endometriosis, myoma, and ovarian cyst	Not reported	Not having taken any medications or vitamin and mineral supplements during three menstrual cycles before the trial	Not reported	Having regular menstrual cycles of 24-35 days	Menstruation cramps of at least 3-5 (moderate to very severe) by menstrual Symptom Questionnaire	Not being professional athletes; no leisure time exercises in the recent three months
Yang (2016)	Self-reported dysmenorrhea	Excluded diagnosis of pelvic or gynecological diseases or secondary dysmenorrhea	Not reported	Not reported	Excluded use of contraceptives	Menstrual cycle duration between 20 and 40 days	Menstrual cramp pain score above 6/10 on VAS	Excluded experience with yoga exercises
Yilmaz (2019)	Self-reported dysmenorrhea	Excluded endometriosis, polycystic ovary syndrome	Not reported	Not reported	Excluded oral contraceptive use	Regular menstrual cycles	Not reported	Excluded regular exercise
Yonglitthipagon (2017)	Self-reported dysmenorrhea	Excluded history of secondary dysmenorrhea	Not reported	Not reported	Not reported	Not reported	Pain score on VAS of 4-7/10	Not reported
Zaid (2022)	WaLIDD Score of ≤ 7	Excluded history of secondary caused of dysmenorrhea or other gynaecological problems	Never gave birth	Excluded those currently on medications (paracetamol, analgesics etc.)	Excluded oral contraception pills	Regular menstrual cycles were included	WaLIDD Scale Score of ≤ 7	Physically inactive were included
