# Supplementary material for: Effectiveness of Therapeutic Exercise in Reducing the Severity of Primary Dysmenorrhea and Associated Symptoms: A Systematic Review and Meta-Analysis
Source: J Clin Med. 2026 Jun 7;15(12):4418. doi: 10.3390/jcm15124418 (PMC13301083; doi:10.3390/jcm15124418)
Supplement: Supplementary file 1 [file jcm-15-04418-s001.zip › Supplementary S4.pdf]

Supplementary appendix 4_ Sensitivity analysis

Table S1_1: Sensitivity analysis for symptom severity outcome

Study excluded	SMD	Lower limit	Upper limit
Akbas et al. 2019	-0.94	-1.40	-0.48
Elbandrawy et al. 2021_aerobic	-0.97	-1.43	-0.52
Huang et al. 2022	-0.88	-1.32	-0.43
Pio-Soria et al. 2025	-0.94	-1.39	-0.49
Vaziri et al. 2015_aerobic	-0.91	-1.36	-0.45
Yilmaz et al. 2019	-0.99	-1.43	-0.55
Aksu et al. 2024	-0.79	-1.18	-0.40
Yang et al. 2016	-0.81	-1.23	-0.40
Elbandrawy et al. 2019_strength	-0.98	-1.43	-0.53
Gim et al. 2018	-0.96	-1.41	-0.51
Öz et al. 2026_strength	-0.90	-1.35	-0.45
Ibrahim et al. 2023_supervised	-0.82	-1.24	-0.40
Ibrahim et al. 2013_unsupervised	-0.93	-1.38	-0.48
Öz et al. 2026_stretch	-0.85	-1.29	-0.42
Vaziri et al. 2015_stretch	-0.97	-1.43	-0.51
Kirmizigil et al. 2020	-0.90	-1.35	-0.45
Koçak et al. 2025	-0.97	-1.43	-0.51

Table S1_2: Sensitivity analysis for pain intensity outcome

Study excluded	MD	Lower limit	Upper limit
Elbandrawy et al. 2021_aerobic	-1.94	-2.36	-1.52
Huang et al. 2022	-2.00	-2.42	-1.57
Pio-Soria et al_2025	-2.04	-2.46	-1.61
Samy et al. 2019	-2.00	-2.43	-1.57
Yilmaz et al. 2019	-2.03	-2.46	-1.60
Chen et al. 2019	-2.07	-2.50	-1.65
Ibrahim et al. 2023_supervised	-2.01	-2.43	-1.58
Ibrahim et al. 2023_unsupervised	-2.06	-2.49	-1.64
Jaibunnisha et al. 2019	-2.09	-2.52	-1.65
Mirzaei et al. 2021	-2.09	-2.52	-1.66
Öz et al. 2026_stretch	-2.00	-2.42	-1.57
Azima et al. 2015	-2.03	-2.46	-1.60
Elbandrawy et al. 2021_strength	-1.95	-3.28	-1.73
Gim et al. 2018	-2.13	-2.54	-1.72
Öz et al. 2026_strength	-2.02	-2.44	-1.60
Shahrjerdi et al. 2019	-2.00	-2.44	-1.56
Zaid et al. 2022	-2.04	-2.46	-1.62
Aksu et al. 2024	-1.93	-2.35	-1.51
Kirca et al. 2023	-2.12	-2.53	-1.71
Rakhshaee et al. 2011	-2.09	-2.52	-1.66
Şaşmaz et al. 2024	-2.05	-2.48	-1.62
Silwal et al. 2025	-2.07	-2.49	-1.64
Yang et al. 2016	-2.09	-2.51	-1.66
Yonglitthipagon et al. 2017	-2.04	-2.47	-1.62
Abbas et al. 2023	-2.05	-2.47	-1.62
Heidarimoghadam et al. 2019	-2.05	-2.48	-1.63
Kirmizigil et al. 2020	-2.10	-2.52	-1.68
Ortiz et al 2015	-2.03	-2.52	-1.55

Table S1_3: Sensitivity analysis for pain duration outcome

**Study excluded**	**SMD**	**Lower limit**	**Upper limit**
Samy et al. 2019	0.51	0.006	0.95
Azima et al. 2015	0.58	0.40	1.36
Shahrjerdi et al. 2019	0.69	0.08	1.30
Zaid et al. 2022	0.61	0.04	1.18
Rakhshaee et al. 2011	0.77	0.12	1.42
Yang et al. 2016	0.78	0.18	1.38
Heidarimoghadam et al. 2019	0.68	0.03	1.34

Table S1_4: Sensitivity analysis for quality-of-life outcome

**Study excluded**	**SMD**	**Lower limit**	**Upper limit**
Akbas et al. 2019	0.77	0.24	1.29
Pio-Soria et al. 2025	0.78	0.28	1.28
Temizkan et al. 2025	0.74	0.22	1.27
Öz et al. 2026_strength	0.64	0.14	1.14
Öz et al. 2026_stretch	0.63	0.14	1.12
Aksu et al. 2024	0.60	0.13	1.07
Şaşmaz et al. 2024	0.79	0.28	1.31
Silwal et al. 2025	0.81	0.32	1.30
Yonglitthipagon et al. 2017	0.58	0.12	1.04

Table S1_5: Sensitivity analysis for anxiety outcome

**Study excluded**	**SMD**	**Lower limit**	**Upper limit**
Akbas et al. 2019	0.66	0.03	1.28
Azima et al. 2015	0.49	-0.52	1.50
Huang et al. 2022	0.13	-0.37	0.63
Pio-Soria et al. 2025	0.35	-0.43	1.13

Table S1_6: Sensitivity analysis for sleep quality outcome

**Study excluded**	**SMD**	**Lower limit**	**Upper limit**
Huang et al. 2022	0.38	-0.06	0.82
Pio-Soria et al. 2025	0.39	-0.02	0.81
Öz et al. 2026_strength	0.22	-0.10	0.54
Öz et al. 2026_stretch	0.27	-0.09	0.63
Kirmizigil et al. 2020	0.42	-0.01	0.83
Koçak et al. 2025	0.50	0.14	0.87
