# Supplementary material for: Effectiveness of Therapeutic Exercise in Reducing the Severity of Primary Dysmenorrhea and Associated Symptoms: A Systematic Review and Meta-Analysis
Source: J Clin Med. 2026 Jun 7;15(12):4418. doi: 10.3390/jcm15124418 (PMC13301083; doi:10.3390/jcm15124418)
Supplement: Supplementary file 1 [file jcm-15-04418-s001.zip › Supplementary S5.pdf]

Supplementary appendix S5_Subgroup analysis

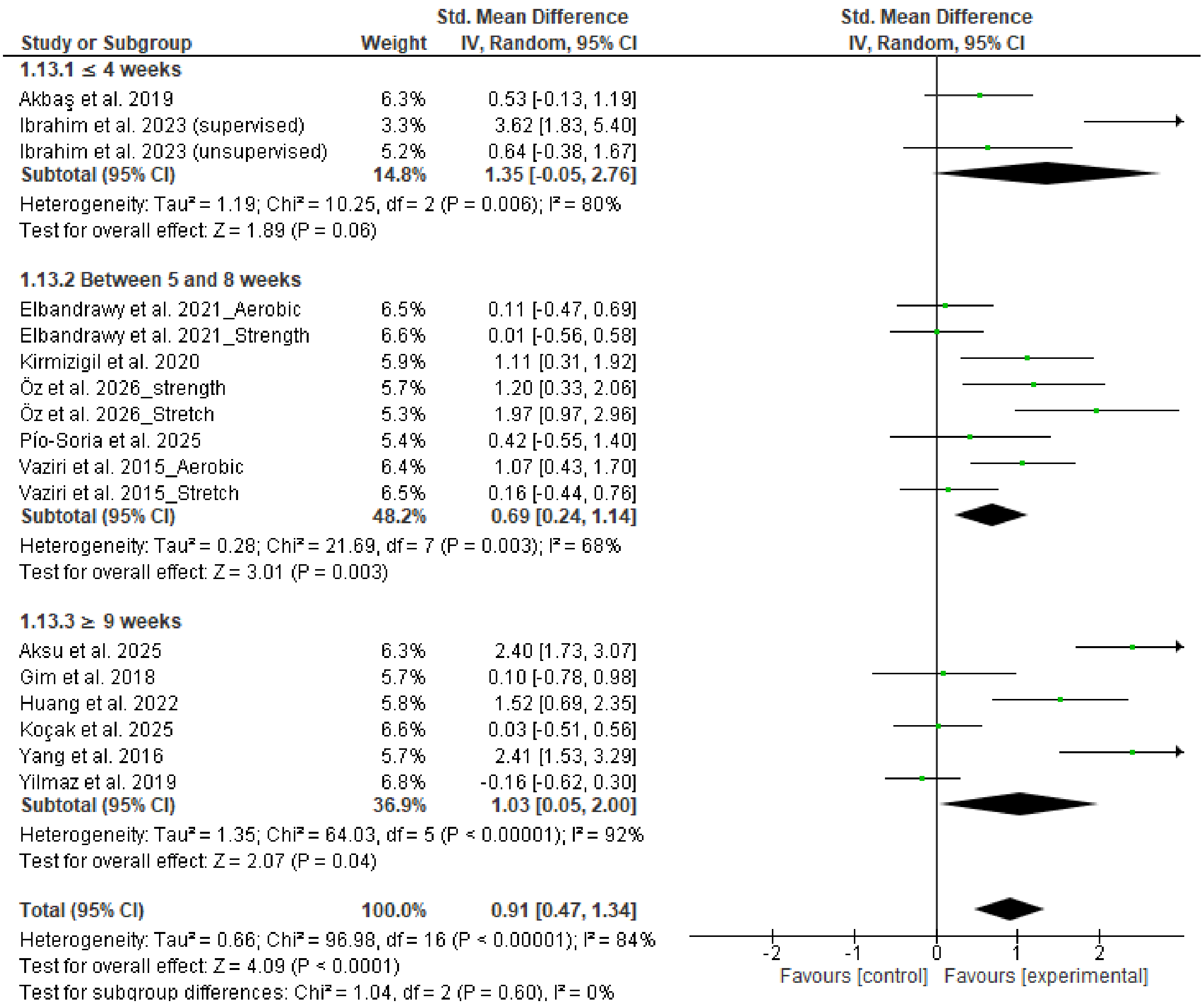

Figure S1_1: Effect of TE on symptom severity after intervention depending on the duration of the programs. The green dots and black horizontal lines show effect estimates and 95% confidence intervals, respectively.

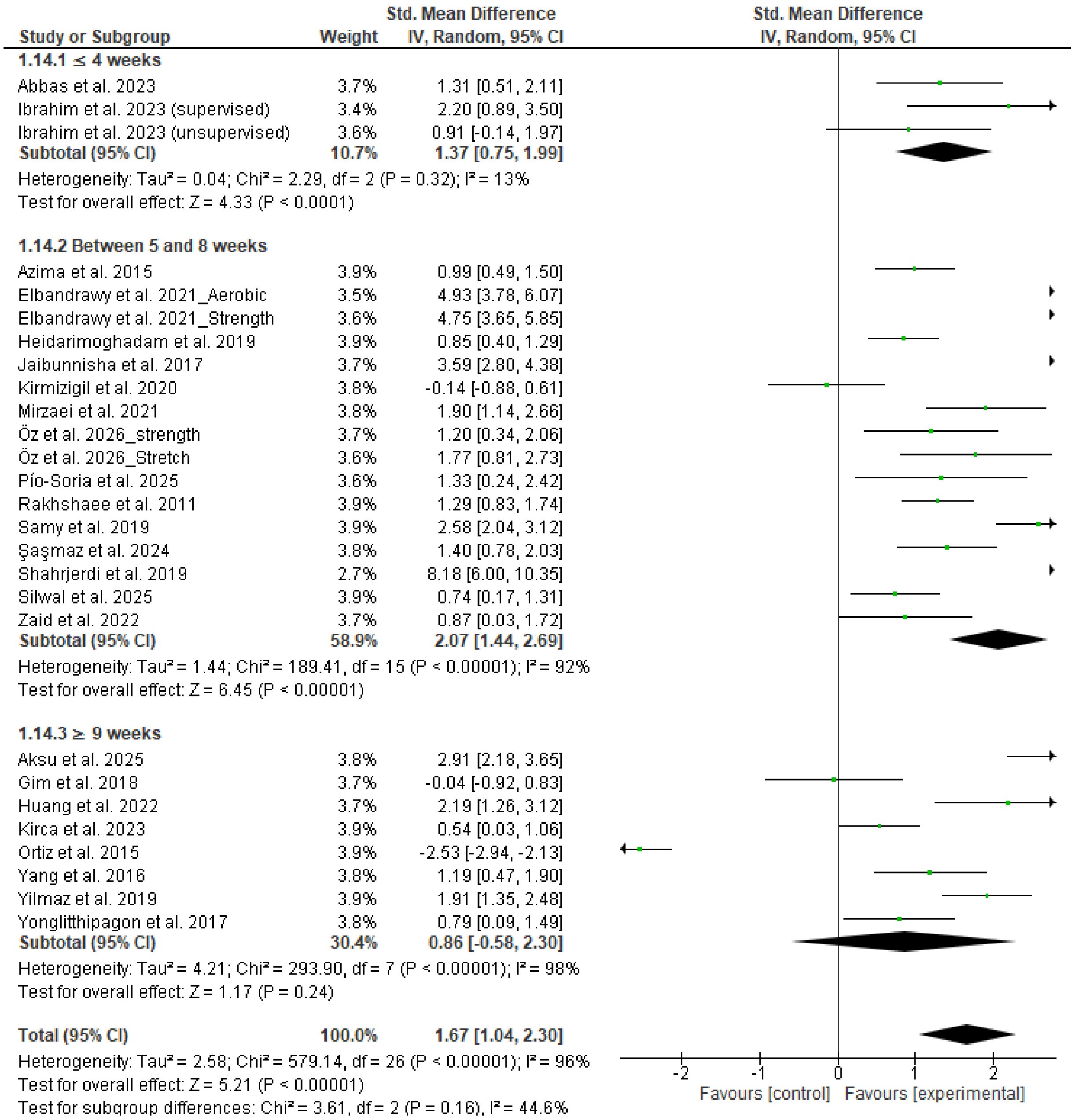

Figure S1_2: Effect of TE on pain intensity after intervention depending on the duration of the programs. The green dots and black horizontal lines show effect estimates and 95% confidence intervals, respectively.

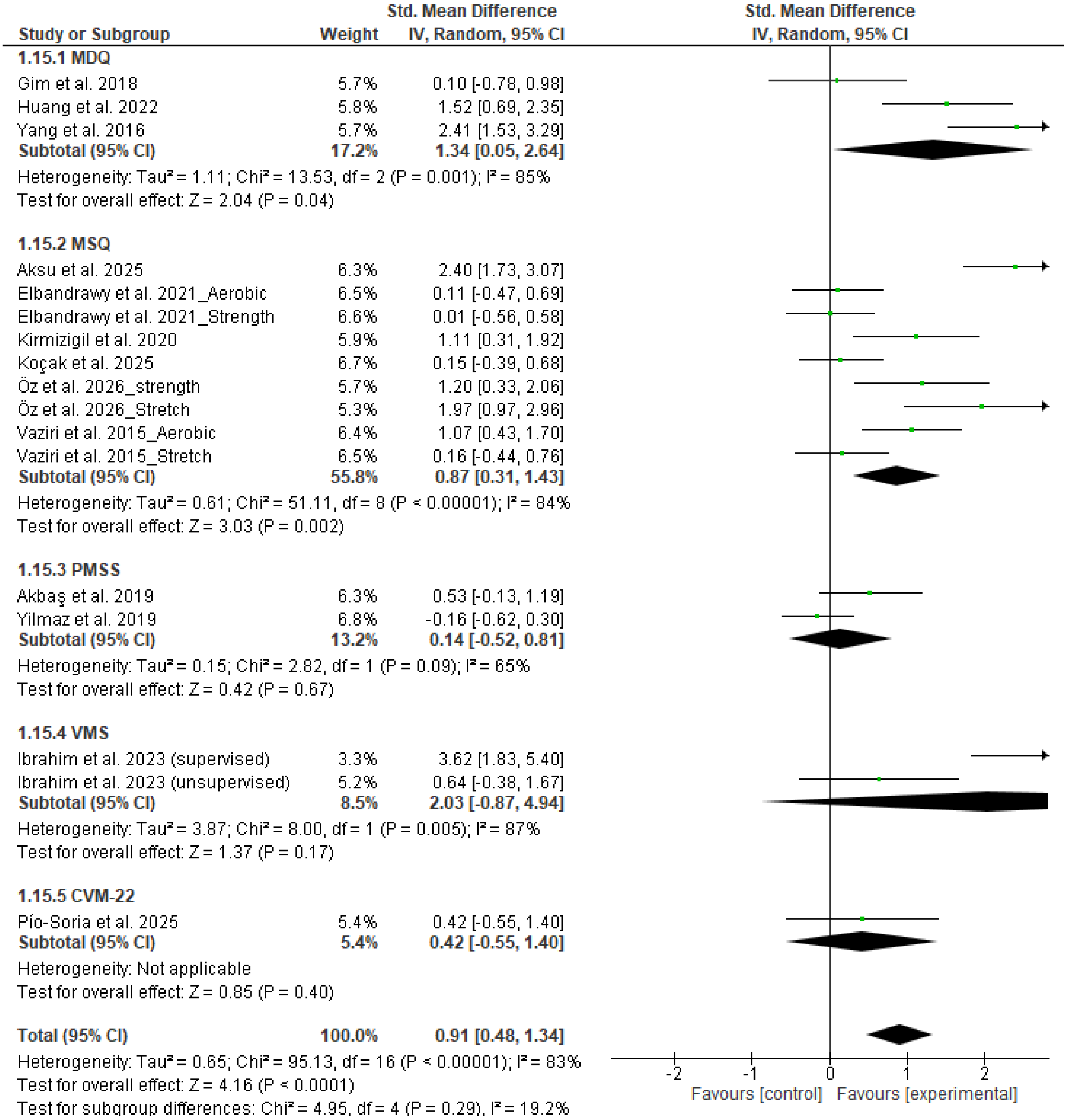

Figure S1_3: Effect of TE on symptom severity after intervention depending on the questionnaire used for the assessment. The green dots and black horizontal lines show effect estimates and 95% confidence intervals, respectively.

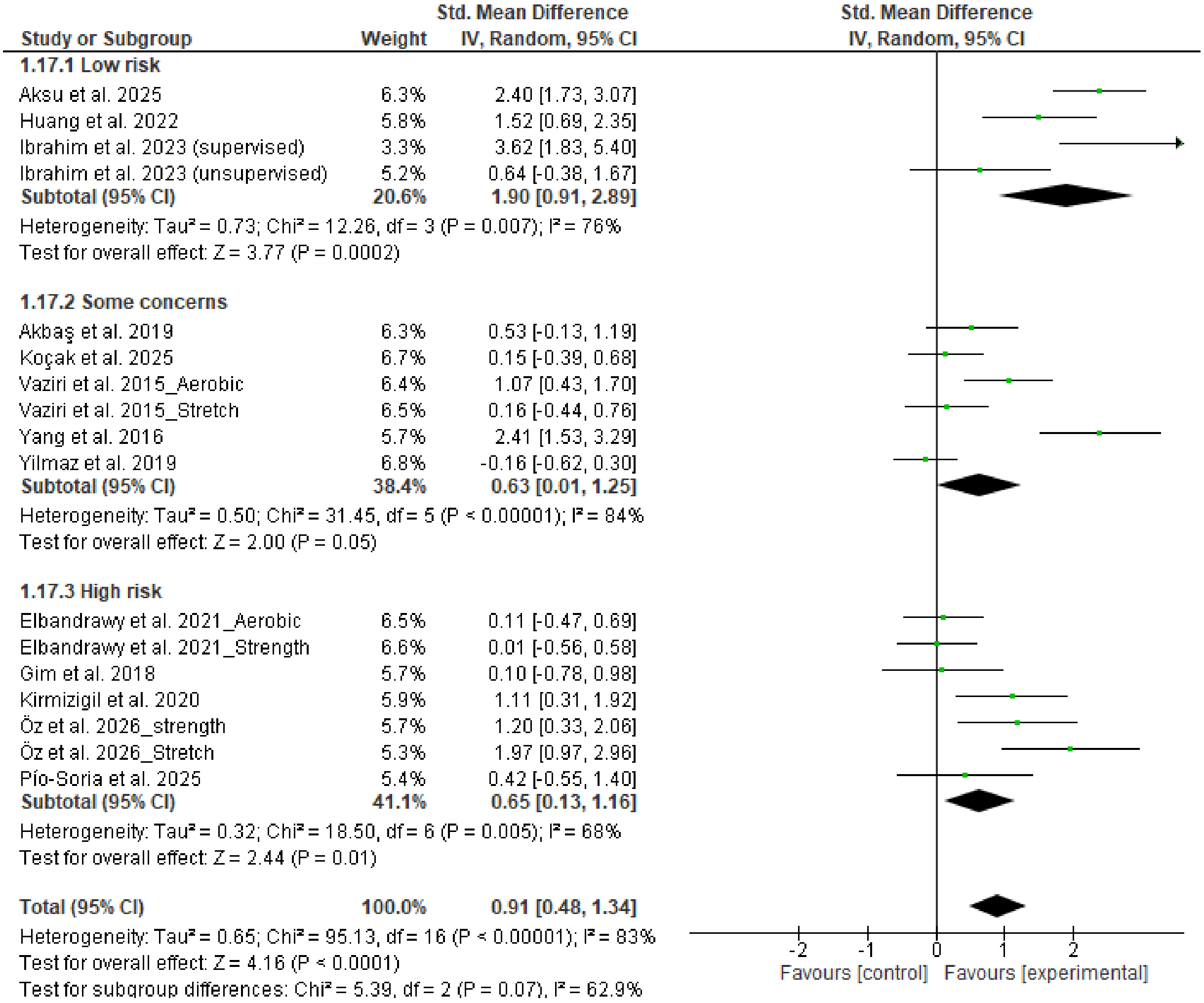

Figure S1_4: Effect of TE on symptom severity after intervention depending on the risk of bias assessment. The green dots and black horizontal lines show effect estimates and 95% confidence intervals, respectively.

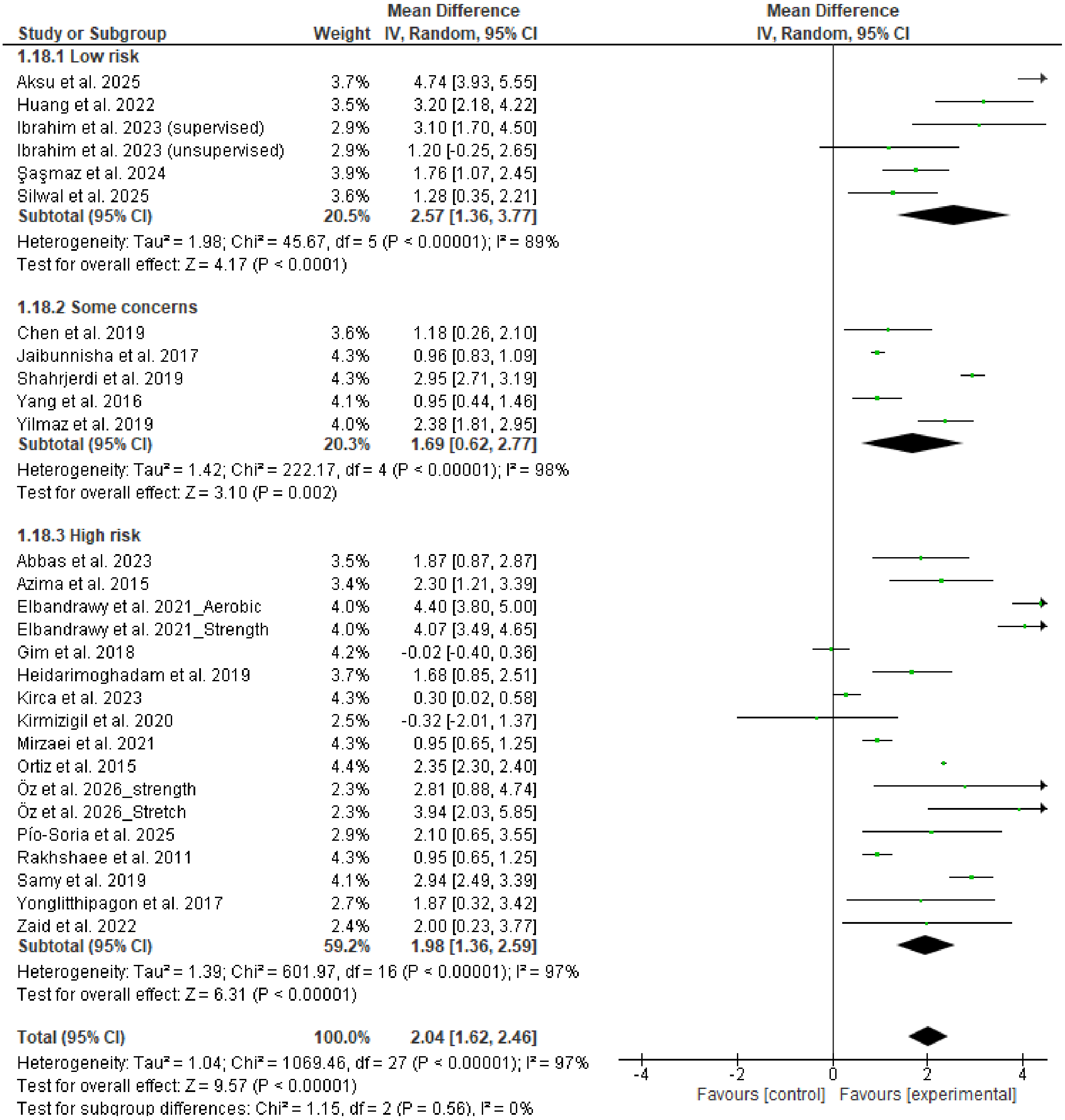

Figure S1_5: Effect of TE on pain intensity after intervention depending on the risk of bias assessment. The green dots and black horizontal lines show effect estimates and 95% confidence intervals, respectively.

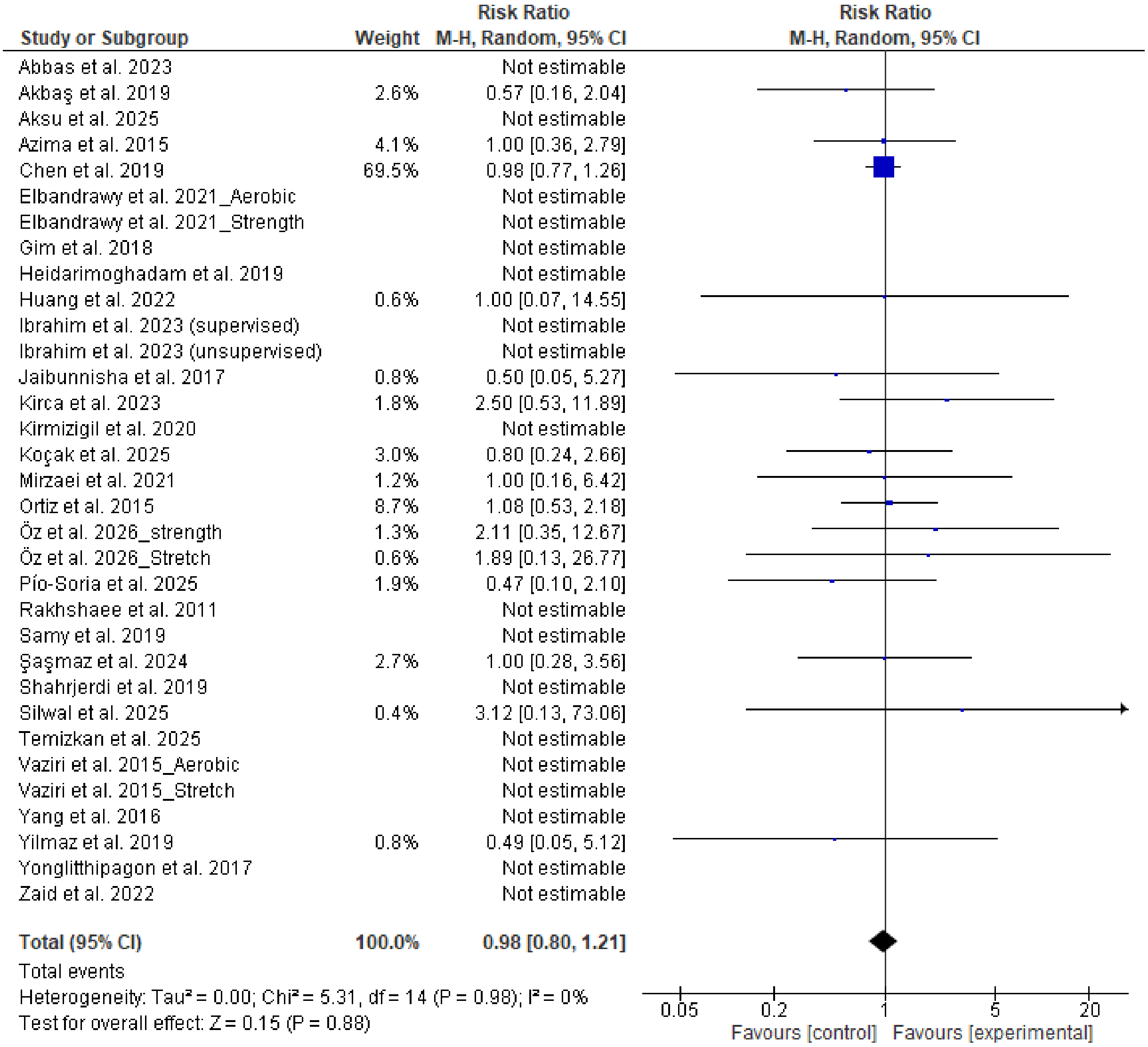

Figure S1_6: Dropout ratio between the intervention group and the control group for each study.
