# Supplementary material for: Effectiveness of Therapeutic Exercise in Reducing the Severity of Primary Dysmenorrhea and Associated Symptoms: A Systematic Review and Meta-Analysis
Source: J Clin Med. 2026 Jun 7;15(12):4418. doi: 10.3390/jcm15124418 (PMC13301083; doi:10.3390/jcm15124418)
Supplement: Supplementary file 1 [file jcm-15-04418-s001.zip › Supplementary S6.pdf]

Supplementary appendix S6_Funnel plots of the main outcomes

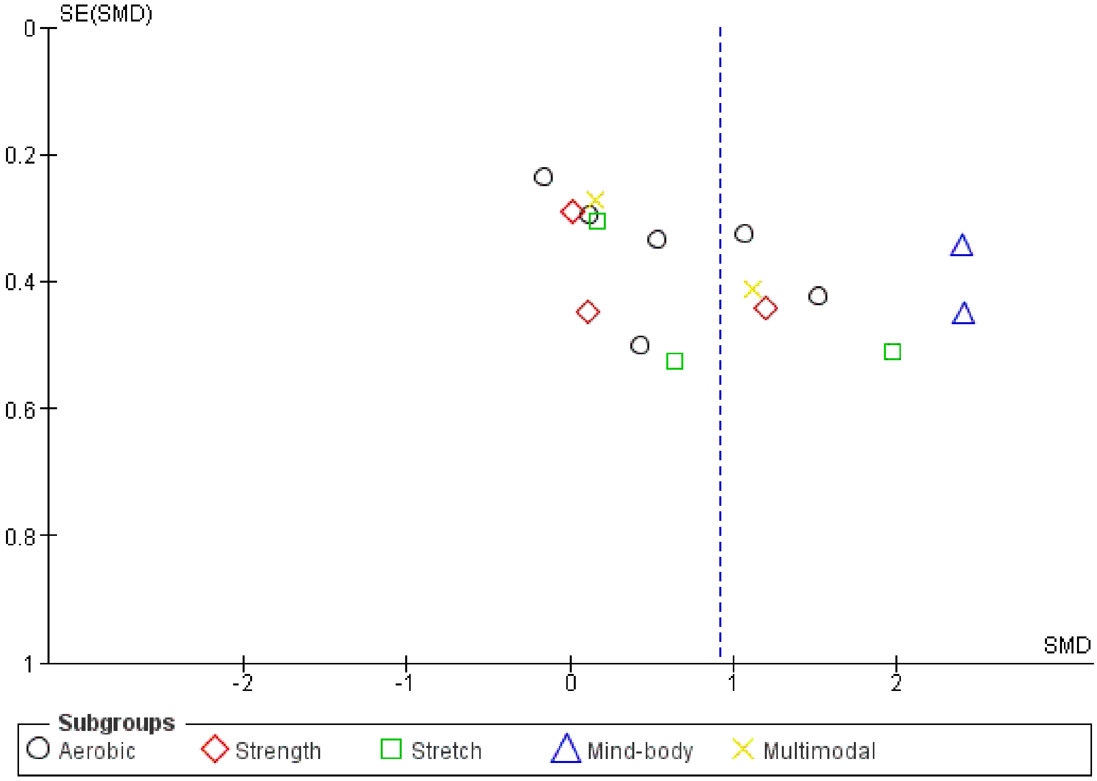

Figure S2_1: Funnel plot of symptom severity outcome.

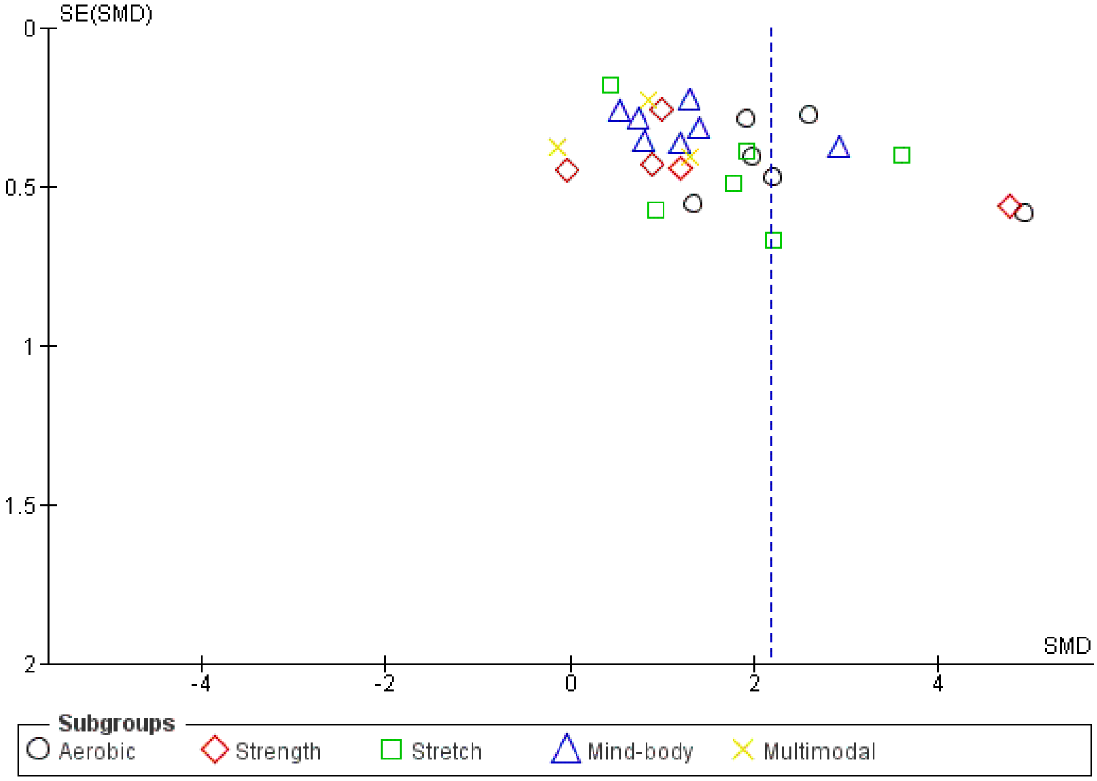

Figure S2_2: Funnel plot of pain intensity outcome.
